# Highly active deficient ternary sulfide photoanode for photoelectrochemical water splitting

**DOI:** 10.1038/s41467-020-16800-w

**Published:** 2020-06-17

**Authors:** Haimei Wang, Yuguo Xia, Haiping Li, Xiang Wang, Yuan Yu, Xiuling Jiao, Dairong Chen

**Affiliations:** 1 School of Chemistry and Chemical Engineering, Shandong University, 250100 Jinan, Shandong China; 2 National Engineering Research Center for Colloidal Materials, School of Chemistry and Chemical Engineering, Shandong University, 250100 Jinan, Shandong China; 3 School of Microelectronics, Shandong University, 250100 Jinan, Shandong China

**Keywords:** Electrocatalysis, Artificial photosynthesis, Photocatalysis, Nanoparticles

## Abstract

The exploration of photoanode materials with high efficiency and stability is the eternal pursuit for the realization of practically solar-driven photoelectrochemical (PEC) water splitting. Here we develop a deficient ternary metal sulfide (CdIn_2_S_4_) photoanode, and its PEC performance is significantly enhanced by introducing surface sulfur vacancies, achieving a photocurrent density of 5.73 mA cm^−2^ at 1.23 V vs. RHE and 1 Sun with an applied bias photon-to-current efficiency of 2.49% at 0.477 V vs. RHE. The experimental characterizations and theoretical calculations highlight the enhanced effect of surface sulfur vacancies on the interfacial charge separation and transfer kinetics, which also demonstrate the restrained surface states distribution and the transformation of active sites after introducing surface sulfur vacancies. This work may inspire more excellent work on developing sulfide-based photoanodes.

## Abstract (web summary)

While water splitting could provide a means to utilize solar energy, identifying sufficiently stable and active semiconductors is challenging. Here, authors report a deficient CdIn_2_S_4_ photoanode with improved water splitting performances due to efficient charge separation/transfer kinetics.

## Introduction

The efficient utilization of solar energy may afford a renewable route to alleviate the energy and environmental issues caused by the increasing consumption of fossil fuels^1^. Apart from photovoltaics (PV), which convert solar energy into electricity, photoelectrochemical (PEC) water splitting is recognized as another promising strategy and attracts particular interest for storing solar energy into the chemical bonds of hydrogen fuel, which can be further released and utilized in fuel cells or internal combustion engines^2,3^. To achieve high solar to hydrogen (STH) conversion efficiencies in practical PEC cells, the choice of electrode materials, especially the photoanodes, is of great importance in the consideration of the sluggish, multistep, proton-coupled electron transfer kinetics of water oxidation on photoanodes surfaces^4^. Up to now, some of the most promising semiconductors employed as photoanodes include α-Fe_2_O_3_^5^, BiVO_4_^6^, Ta_3_N_5_(TaON)^7,8^, and TiO_2_^9^. However, no semiconductor so far fully satisfies all the stringent requirements for practical application, including the high STH efficiency, robust stability in aqueous electrolytes, and low cost for scalable module manufacturing, which renders the design of satisfactory photoanode materials still challenge.

Other than making efforts on the current photoanodes materials, exploring novel semiconductors and employed as photoanodes maybe supply a new route. The optimal tandem PEC cell ideally is consisted of a photoanode with a bandgap of ~2.0 eV and a photocathode with a bandgap of ~1.2 eV to achieve the highest STH efficiency and unassisted water splitting^10^. Therefore, the semiconductors that possess proper bandgaps and band-edge potentials theoretically have the potential to be employed as photoanodes. Among others, metal sulfides have long been the topic of research, especially photocatalytic applications due to their suitable electronic bandgaps, exposed active sites, diverse and adjustable chemical structures^11^. However, apart from the factor of photochemical photocorrosion, which can be dramatically suppressed by the usage of sacrificial agents or surface decoration of passive layers^12–14^, the metal sulfides directly employed as photoanodes usually exhibit low photoinduced electron–hole separation efficiencies and sluggish surface water oxidation kinetics^15–18^, which limits the application of metal sulfides-based photoanodes. The defects in metal sulfide, especially for introducing sulfur vacancies, are evidenced as an effective strategy to enhance the photocatalytic and PEC properties^19–22^. The trap states introduced by sulfur vacancies can act as capture centers to enable charge carrier separation. Besides, the charge density of metal sulfide can also be significantly increased after introducing sulfur vacancies, which results in the shortening of depletion width and enhancement for the band bending for hole collection^23^. Moreover, recent research reveals that the adjacent atoms after introducing sulfur vacancies turn into the active sites for oxygen evolution reaction (OER), which can facilitate the surface water oxidation kinetics^24^. Benefit from all these reasons, we consider that introducing sulfur vacancies into metal sulfides-based photoanodes may also be an efficient strategy to improve their PEC performance.

Herein, we develop a deficient ternary metal sulfide (CdIn_2_S_4_) and employ it as a photoanode. We introduce sulfur vacancies to the CdIn_2_S_4_ photoanode through mild annealing treatment, and the PEC performance of the CdIn_2_S_4_ photoanode is significantly enhanced, revealing superior photocurrent density and applied bias photon-to-current efficiency (ABPE) compared to other up-to-date promising single photon absorbers. In addition, the effects of sulfur vacancies on the PEC performance and charge transfer kinetics are systematically elucidated by experimental characterizations and theoretical calculations. Specifically, the surface sulfur vacancies can restrain the surface states (SS) distribution on the CdIn_2_S_4_ photoanode, which supplies an approach to adjusting the potentials at the semiconductor/electrolyte interface.

## Results

### Synthesis and physicochemical characterizations

**Fig. 1:**
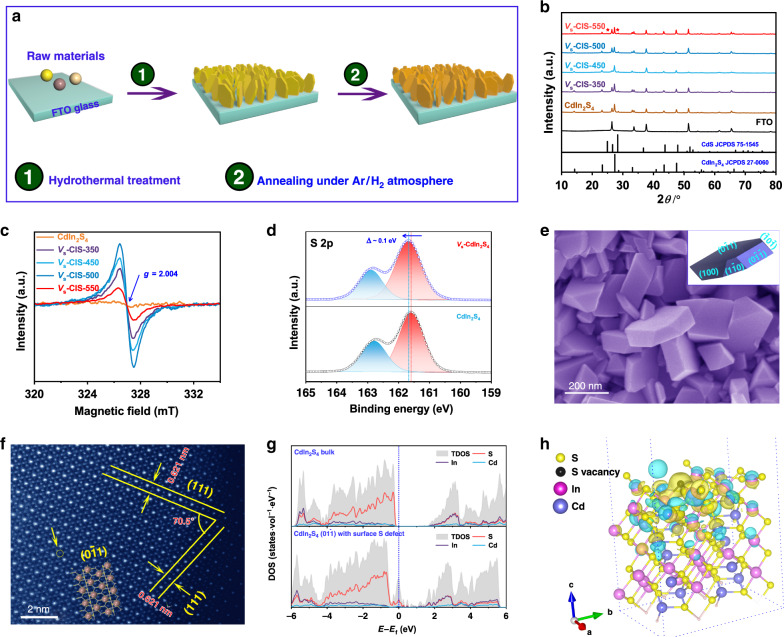
Synthesis and characterizations of S deficient CdIn_2_S_4_ nanocrystals. **a** The schematic diagram for the synthetic procedure of the CdIn_2_S_4_ nanocrystals with sulfur vacancies. **b** PXRD patterns. **c** ESR spectra. **d** Core-level XPS spectra of S 2*p* for CdIn_2_S_4_ and *V*_s_-CIS-500. **e** The SEM image of *V*_s_-CIS-500. Inset is the reconstructed shape based on the BFDH theory. **f** The HADDF-STEM image of *V*_s_-CIS-500. Inset is the atomic structure of CdIn_2_S_4_ ($0\overline 1 1$) with In and S as truncated atoms. **g** The density of states for CdIn_2_S_4_ bulk and CdIn_2_S_4_ ($0\overline 1 1$) with surface sulfur vacancies. **h** Different charge density image for the CdIn_2_S_4_ ($0\overline 1 1$) with surface sulfur vacancies (isosurface set at 0.04e Bohr^−3^); yellow and cyan regions represent electron accumulation and depletion, respectively.

The synthetic protocol of the CdIn_2_S_4_ photoanode with sulfur defects is illustrated in Fig. 1a, which involves two steps, i.e., hydrothermal treatment and subsequent annealing with an Ar/H_2_ flow. Introducing heteroatoms or vacancies is usually more easily achieved under higher temperatures^25^. Therefore, the phase stability of the CdIn_2_S_4_ under different annealing temperatures is first investigated. The powder X-ray diffraction (PXRD) patterns of the final products are shown in Fig. 1b and all the diffractions are well indexed to cubic CdIn_2_S_4_ and FTO except for the product annealed at 550 °C, where the impurity phase of CdS appears as labeled by the asterisks. The evolution of the atomic coordination environment involved in as-prepared products at different temperatures is monitored using electron spin resonance (ESR) spectra (Fig. 1c), which yields a *g* value of 2.004 that is assigned to the sulfur vacancy^26^. The upshift of S 2*p* X-ray photoelectron spectra (XPS) of *V*_s_-CIS-500 further confirms the appearance of sulfur vacancy (Fig. 1d)^24^, which is also validated by the Cd 3*d* and In 3*d* peaks shifting to lower binding energy to compensate the charge nonequilibrium (Supplementary Fig. 1). The peak position of O 1*s* in *V*_s_-CIS-500 is nearly identical to that in pristine CdIn_2_S_4_, which illustrates the existence form of O should be surface hydroxyls (Supplementary Fig. 2). Besides, apart from the intensities of the ESR signals, the photocurrent of the products also reveals a positive correlation with the temperature but *V*_s_-CIS-550 (Supplementary Fig. 3). Thus, we consider that the *V*_s_-CIS-500 maybe possess the optimal PEC performance. The microstructure of *V*_s_-CIS-500 is investigated by scanning electron microscopy (SEM, Fig. 1e), which exhibits plate-shaped geometry and reveals nearly no changes compared with that of the pristine CdIn_2_S_4_ nanocrystals (Supplementary Fig. 4), indicating that the introduction of sulfur vacancies does not affect its morphology. The energy-disperse X-ray spectroscopy (EDS, Supplementary Fig. 5) confirms that the Cd, In, and S atoms distribute uniformly across the *V*_s_-CIS-500 nanoplate, also indicative of its structural stability during annealing treatment. Besides, the morphology of *V*_s_-CIS-500 is reconstructed according to the Bravais–Friedel–Donnay–Harker (BFDH) theory (inset of Fig. 1e)^27^, and further surface energy calculations reveal that the ($0\overline 1 1$) crystal plane truncated with In and S atoms is the dominant exposed plane (see details in Supplementary Note 1). The formation energies of sulfur vacancies in all possible chemical potentials are calculated as well, which illustrate that the formation of sulfur vacancies is a thermodynamically endothermic process and ($0\overline 1 1$) is energetically the most feasible crystal plane to generate surface sulfur vacancies under all possible chemical potentials (see details in Supplementary Note 2). Thus, we consider that surface sulfur vacancies in the ($0\overline 1 1$) crystal plane is mainly responsible for the PEC performance changes of the CdIn_2_S_4_ photoanode. The selected area electron diffraction (SAED, Supplementary Fig. 6) illustrates the single-crystal characteristic of *V*_s_-CIS-500, and the high-angle annular dark-field (HAADF) image (Fig. 1f) further confirms the dominant ($0\overline 1 1$) crystal plane, as well as shows the existence of surface sulfur vacancies as marked by the circle. Moreover, the theoretical atomic structure of ($0\overline 1 1$) truncated with In and S atoms matches well with the HAADF-STEM image (inset of Fig. 1f), which also corresponds to above surface energy calculation results. To further elucidate the effect of surface sulfur vacancies on the electronic structures of CdIn_2_S_4_ nanocrystals, we performed Coulomb interaction corrected density functional theory (DFT + *U*) calculations. As depicted in Fig. 1g, shallow trap states mainly consisted of S 3*p* orbitals generate, which maybe contributes to enhanced photoabsorption or improve interfacial electron transfer processes. More importantly, the generation of surface sulfur vacancies results in the charge accumulation on the adjacent Cd and In atoms (Fig. 1h), which may act as highly active sites for chemisorption of the intermediates during the OER, and facilitates the surface water oxidation kinetics.

### PEC performance of CdIn_2_S_4_ and *V*_s_-CIS-500 photoanodes

**Fig. 2:**
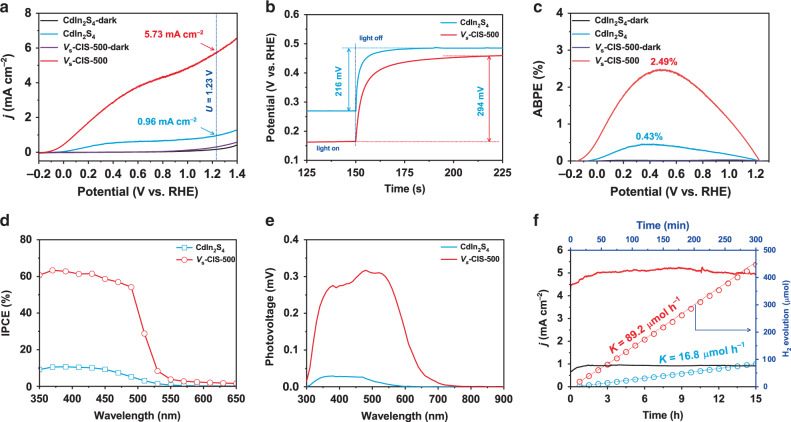
Photoelectrochemical performance. **a** Current–voltage (*J–V*) plots measured in 0.35 M Na_2_SO_3_ and 0.25 M Na_2_S mixed solution (pH = 12.5) under dark and AM 1.5 G, 100 mW cm^−2^ illumination with a scan rate of 10 mV s^−1^. **b** Open-circuit voltages of CdIn_2_S_4_ and *V*_s_-CIS-500 photoanodes were recorded in 0.5 M Na_2_SO_4_ solution (pH = 7) in the dark and under illumination. **c** ABPE measured with a two-electrode cell for water splitting in 0.35 M Na_2_SO_3_ and 0.25 M Na_2_S mixed solution (pH = 12.5) under dark and AM 1.5 G, 100 mW cm^−2^ illumination. **d** IPCE measured at 0.6 V vs. RHE under monochromatic light irradiation. **e** SPV spectra of CdIn_2_S_4_ and *V*_s_-CIS-500 nanoparticles. **f** Long-term stability test, as well as experimental and theoretical H_2_ evolution, were measured in 0.35 M Na_2_SO_3_ and 0.25 M Na_2_S mixed solution (pH = 12.5) under AM 1.5 G, 100 mW cm^−2^ illumination.

The PEC performance of CdIn_2_S_4_ and *V*_s_-CIS-500 photoanodes was investigated with a conventional three-electrode cell. The *J–V* plots obtained using AM 1.5 G illumination reveal that the photocurrent density of the *V*_s_-CIS-500 photoanode is greatly enhanced, achieving a value of 5.73 mA cm^−2^ at 1.23 V vs. RHE, and corresponds to a ~6 times increase compared with that of the pristine CdIn_2_S_4_ photoanode (Fig. 2a). The *V*_s_-CIS-500 photoanode reveals the generation of photocurrent density higher than 5.0 mA cm^−2^ at 1.23 V vs. RHE without OER cocatalysts, which is superior to most of the other metal sulfide-based photoanodes (Supplementary Table 1) and also comparable to other up-to-date promising photoanodes, such as α-Fe_2_O_3_, BiVO_4_, and Ta_3_N_5_ (Supplementary Table 2). The onset potential (*V*_on_) is determined by the intersection point of the *J–V* plot subtracting the contribution of the dark current curve (Supplementary Fig. 7). Noteworthily, the onset potential of the *V*_s_-CIS-500 photoanode (−148 mV) reveals a cathodic shift of 74 mV relative to that of CdIn_2_S_4_ (−74 mV). The onset potential of the photoanode under illumination is generally influenced by two factors, i.e., the open-circuit photovoltage (*V*_ph_) and the kinetic overpotential (*η*_k_) as expressed by the following equation: *E*_redox_–*V*_onset_ = *V*_ph_–*η*_k_, where *E*_redox_ represents the electrochemical potential of the electrolyte solution that is irrelevant to the surface nature of the electrode^28^. Given that either an increase of *V*_ph_ or a decrease of *η*_k_ can give rise to the cathodic shift of *E*_onset_, confirming the crucial factor is of great importance to understand the effect of surface sulfur vacancies. The difference between the quasi-equilibrium under illumination and the equilibrium for CdIn_2_S_4_ and *V*_s_-CIS-500 photoanodes is reported on the photovoltage (Fig. 2b, Supplementary Fig. 8). The difference of 78 mV between CdIn_2_S_4_ and *V*_s_-CIS-500 photoanodes accounts for the above cathodic shift of 74 mV, illustrating that the increase of *V*_ph_ rather than the decrease of *η*_k_ is mainly responsible for the observed cathodic shift. Meanwhile, the equilibrium open-circuit potentials of both CdIn_2_S_4_ (0.485 ± 0.003 V) and *V*_s_-CIS-500 (0.458 ± 0.004 V) photoanodes in the dark deviate from the ideal value (1.23 V), indicative of the significant potential drop within their Helmholtz layers^29^, which is detrimental to the photovoltage generation capabilities of CdIn_2_S_4_ and *V*_s_-CIS-500 photoanodes. In principle, this adverse potential drop can be restrained by modification of co-catalysts^30,31^, which, however, is beyond the scope of this manuscript and will be discussed in our future work. The ABPE values derived from the *J–V* plots are also calculated (Eq. (2)), and the *V*_s_-CIS-500 photoanode reveals a record-high value for single sulfide photon absorber to date (Fig. 2c), achieving the maximum ABPE value of 2.49% at 0.477 V vs. RHE (Supplementary Table 1).

Meanwhile, the incident photon-to-current conversion efficiencies (IPCE) under monochromatic light irradiation are also employed to illustrate the photoresponse of CdIn_2_S_4_ and *V*_s_-CIS-500 photoanodes (Fig. 2d, Eq. (3)), which reveals that the IPCE value is significantly improved in the light range of 350–500 nm, and the light to generate measurable photovoltage is truncated between 525 and 550 nm for the CdIn_2_S_4_ photoanode while it extends up to ~650 nm for the *V*_s_-CIS-500 photoanode. To further verify whether the photocurrent enhancement is solely due to the improved photon absorption, we further calculate the absorbed photon-to-current efficiency (APCE, Supplementary Fig. 9), which manifests a significant enhancement in APCE for the *V*_s_-CIS-500 photoanode, particularly from 350 to 500 nm, indicating the improved efficiency in electron–hole separation. To further unravel the effect of surface sulfur vacancies on the electron–hole separation efficiency in the *V*_s_-CIS-500 photoanode, the surface photovoltage (SPV) spectroscopy was conducted (Fig. 2e). The positive SPV spectra confirm the type of SS, that is, excitation of electrons from the SS to the conduction band^32^. Of note, except for the significantly increased photovoltage for the *V*_s_-CIS-500 photoanode which demonstrates its enhanced electron–hole separation, a broad peak is detected, and distinct sub-bandgap knees emerge, which is caused by both the Franz–Keldysh effect and photo-assisted charge transfer originated from shallow trap states^33^, also illustrative of the existence of trap states generated by sulfur vacancies. The PEC stability is examined by long-term photocurrent to time (*J*–*t*) measurement and the hydrogen evolution test at 0 V vs. Ag/AgCl reference electrode (Fig. 2f). The *J*–*t* plot of *V*_s_-CIS-500 photoanode reveals no significant decay in the 15 h continued operation in 0.35 M Na_2_SO_3_ and 0.25 M Na_2_S-mixed solution, illustrative of the stable characteristic of surface sulfur vacancies under illumination and applied bias. The XRD pattern, SEM image, and ESR signal of the *V*_s_-CIS-500 photoanode post-PEC test are further measured (Supplementary Fig. 10). No phase structural, morphological, and electronic structural changes are observed, indicative of its robust PEC stability. Besides, the XPS spectra of the *V*_s_-CIS-500 photoanode post-PEC test are also measured (Supplementary Fig. 11). The peak positions for Cd and In atoms are nearly unchanged, whereas the S 2*p* orbital slightly shifts to higher binding energy and peaks that assigned to elemental sulfur appear, illustrative of oxidation reaction competing with the consumption rates of photoinduced carriers for water oxidation on the surface of *V*_s_-CIS-500 photoanode^34^, which suggests that the surface of *V*_s_-CIS-500 requires oxygen evolution catalysts (OECs) to form a stable *V*_s_-CIS-500/OEC interface to achieve long-term stability for photo-oxidation of water. Besides, the practical gas evolution in the Pt counter electrode is measured by gas chromatography (GC, Fig. 2f). The hydrogen evolution rate of the *V*_s_-CIS-500 photoanode achieves a release rate of 89.2 μmol h^−1^, which is ~5 times that of the pristine CdIn_2_S_4_ photoanode and approaches its theoretical value (the dashed line). The Faradaic efficiency for hydrogen production measured at 0 V vs. Ag/AgCl reference electrode approaches 100% (Supplementary Fig. 12), also indicative of its high conversion efficiency. Furthermore, the PEC performance of the *V*_s_-CIS-500 photoanode without sacrificial agents are also investigated, revealing a current density of 4.76 mA cm^−2^ at 1.23 V vs. RHE and an ABPE value of 1.35% at 0.733 V vs. RHE (Supplementary Fig. 13), which are greatly enhanced compared with those of the pristine CdIn_2_S_4_ photoanode and also comparable to other up-to-date promising photoanodes (Supplementary Table 2). However, the current density obviously decreases in 1 h continuous measurement in the 0.5 M Na_2_SO_4_ solution, which also illustrates that surface modification of the *V*_s_-CIS-500 photoanode is necessary to stabilize the *V*_s_-CIS-500/electrolyte interface to achieve long-term stability.

### Optical and PEC characterizations for mechanism

**Fig. 3:**
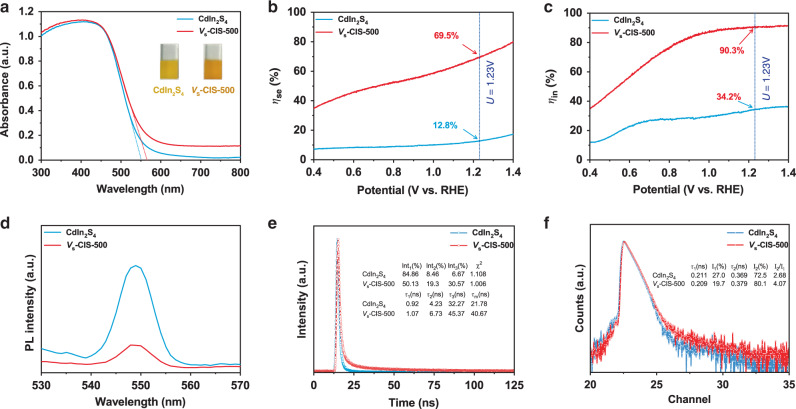
Optical and photoelectrochemical characterizations. **a** UV–vis diffuse reflectance spectra. **b** Charge separation efficiency and **c** charge injection efficiency vs. potential curves. **d** Steady-state PL spectra with excitation wavelength *λ*_ex_ = 370 nm. **e** Time-resolved transient PL decay spectra with excitation wavelength *λ*_ex_ = 370 nm. Inset is the fitted results according to a tri-exponential model, $I\left( t \right) = \sum\nolimits_{i = 1}^{i = 3} {A_i{\mathrm{{e}}}^{ - t/\tau _i}}$, where *I*(*t*), *τ*_i,_ and *A*_i_ were the intensity, the lifetime, and the amplitude of the *i*_th_ component, respectively. **f** PALS of CdIn_2_S_4_ and *V*_s_-CIS-500. Inset is the fitted results according to the following equation, $N\left( t \right) = \mathop {\sum}\nolimits_{{\mathop{i}\nolimits} = 1}^{{\mathop{k}\nolimits} + 1} {\frac{{I_{\mathrm{i}}}}{{\tau _i}}\exp \left( { - \frac{t}{{\tau _i}}} \right)}$, where *τ*_1_*, τ*_2_*, τ*_3_ were three positron lifetime components, and *I*_1_, *I*_2_ *I*_3_ were corresponding to the relative intensities.

The strategies to improve the photoelectric conversion efficiency of the CdIn_2_S_4_ photoanode follow the basic principles as in photocatalyst, that is, to achieve high photoabsorption ability (*J*_abs_), charge separation efficiency (*η*_se_) and injection efficiency (*η*_in_)^35^ (Eq. (4)). Thus, the increase of any term of them can contribute to the final improved PEC performance. To illustrate the effect of sulfur vacancies in the *V*_s_-CIS-500 photoanode and subsequently confirm the crucial factors for the improved PEC activity, we further measure the optical properties. The absorbance edge of *V*_s_-CIS-500 relative to that of CdIn_2_S_4_ slightly extends from ~554 to ~579 nm, indicative of the small change in the bandgap. Meanwhile, the Urbach tail appears due to the shallow trap states generated by sulfur vacancies^36^ (Fig. 3a). The exact energy band structures of *V*_s_-CIS-500 are further determined by ultraviolet photoelectron spectroscopy (UPS) spectra and valence-band XPS spectra (Supplementary Fig. 14), which reveals few changes in the positions of valence band maximum (VBM), the conduction band minimum (CBM), and Fermi level (*E*_f_). Thus, we conclude that the shallow trap states generated by sulfur vacancies rather than the changes of the intrinsic band positions are the critical factors to improve PEC activity. To further elucidate the effect of surface sulfur vacancies, the photon absorption rates of CdIn_2_S_4_ and *V*_s_-CIS-500 photoanodes are calculated (Supplementary Fig. 15, Eq. (5)), and the *J*_abs_ value of *V*_s_-CIS-500 does not reveal much enhancement relative to that of CdIn_2_S_4_, which illustrates that introducing sulfur vacancies cannot improve light harvesting and *J*_abs_ is not the crucial factor for the boosted photocurrent density. The *η*_se_ is related to the photoinduced electron–hole separation, while the *η*_in_ is related to the electron–hole recombination on their surfaces^37^. Both the values of *η*_se_ and *η*_in_ are significantly improved in the *V*_s_-CIS-500 photoanode relative to those in the CdIn_2_S_4_ photoanode (Fig. 3b, c, Eqs. (6) and (7)), illustrating that the more efficient photoinduced electron–hole separation and holes transferring to the surface after introducing of surface sulfur vacancies. Thus, the sulfur vacancies mainly account for the improved *η*_se_ and *η*_in_, which are also crucial factors to the boosted photocurrent.

Moreover, the efficiency of charge separation is investigated by steady-state photoluminescence (PL) spectra (Fig. 3d), and a broad emission peak derived from band-to-band transition is observed at ca. 549 nm. The lower PL peak intensity of *V*_s_-CIS-500 also illustrates its suppressed photoinduced charge recombination that is probably arising from the enhanced charge transport. More importantly, the reduced PL intensity of *V*_s_-CIS-500 demonstrates that the shallow trap states introduced by sulfur vacancies facilitate the photoinduced charge carrier separation rather than recombination. The charge carrier lifetimes of CdIn_2_S_4_ and *V*_s_-CIS-500 are further evaluated by time-resolved photoluminescence (TRPL) spectra (Fig. 3e). The PL decay can be well fitted to a tri-exponential model, and the *V*_s_-CIS-500 manifests a longer average lifetime (40.67 ns) than CdIn_2_S_4_ (21.78 ns), indicating that more photoinduced electrons and holes can participate in the reaction for *V*_s_-CIS-500. Simultaneously, the lifetime of bulk and surface defects can be directly measured by positron annihilation lifetime spectra (PALS) (Fig. 3f). The lifetime components *τ*_1_ and *τ*_2_ correspond to positrons captured by bulk defects and surface defects, respectively^38^. The values of *τ*_1_ and *τ*_2_ for *V*_s_-CIS-500 alter little compared with those of CdIn_2_S_4_, indicative of their similar characteristics for surface and bulk defects^39^. Moreover, The value of *I*_2_/*I*_1_ that reflects the intensity ratio of surface to bulk defects is calculated to be 4.07 for *V*_s_-CIS-500, obviously higher than the 2.68 of pristine CdIn_2_S_4_, which directly demonstrates that the enhanced surface-deficient density is ascribed to the introduction of sulfur surface vacancies.

### Charge transfer and recombination kinetics

**Fig. 4:**
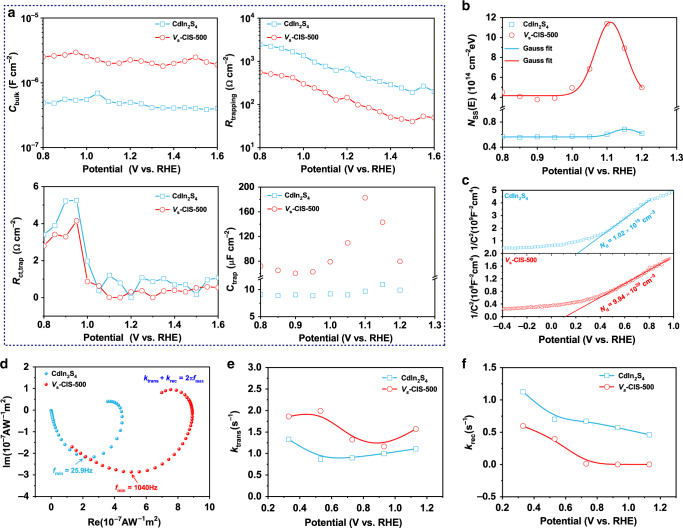
Charge transfer and recombination kinetics. **a** Capacitances and resistances parameters associated with the charge trapping (*R*_trapping_, *C*_bulk_) and transfer (*R*_ct, trap_, *C*_trap_) at/from surface states with different applied potentials that are determined from the EIS spectra. Among others, the *R*_s_ is defined as the series resistance in the PEC cell, *C*_bulk_ represents the capacitance of charge accumulation in the space charge layer, *R*_trapping_ represents the resistance of holes trapping at the surface states, *R*_ct, bulk_ represents the resistance of holes directly transferred from valence band to conduction band, *R*_ct, trap_ represents the resistance of charge transfer from the surface states to the solution, and *C*_trap_ represents the capacitance associated with charge accumulation on the surface states. **b** DOS of surface states (SS) as a function of applied potential for CdIn_2_S_4_ and *V*_s_-CIS-500 photoanode derived from the *C*_trap_. The lines are fitted by a Gaussian model. **c** Mott–Schottky plots. **d** IMPS spectra of the CdIn_2_S_4_ and *V*_s_-CIS-500 photoanodes under the applied bias of 1.13 V. **e** The plot of the rate constant of charge transfer vs. potential. **f** The plot of the rate constant of charge recombination vs. potential.

To more clearly illustrate charge recombination and transfer kinetics, we further investigate the interfacial kinetics of CdIn_2_S_4_ and *V*_s_-CIS-500 photoanodes. There are two different mechanisms for possible hole transfer routes, that is, direct hole transfer from valence band or indirect hole transfer by trapping holes at SS (Supplementary Fig. 16), and the route transferred through SS are recognized as the predominant route, whose density of states (DOS) is proportional to the photocurrent density^40^. Therefore, we employ the electrochemical impedance spectroscopy (EIS) to incisively illustrate changes of the resistances and capacitances that are associated with the charge trapping (*R*_trapping_, *C*_bulk_) and transfer (*R*_ct, trap_, *C*_trap_) at/from SS. The values for the charge trapping and transfer parameters are extracted from the fitted EIS plots according to the corresponding equivalent circuit^41^ (see details in Supplementary Fig. 17). The significant enhanced *C*_bulk_ value for *V*_s_-CIS-500 indicates the increased carrier density after introducing sulfur vacancies in the depletion layer (Fig. 4a), which is consistent with the DOS calculation (Fig. 1g), and the decreased *R*_trapping_ value for *V*_s_-CIS-500 illustrates the recombination of photoinduced electrons and holes are much restrained. Meanwhile, the correlation between the increase of *C*_trap_ and the decrease of *R*_ct, trap_ for the *V*_s_-CIS-500 photoanode confirms that the photo-oxidation indeed occurs from the SS as reported in other works^42,43^. Moreover, compared with the CdIn_2_S_4_ photoanode, the *V*_s_-CIS-500 photoanode reveals more drastic changes in *C*_trap_ rather than in *C*_bulk_, illustrative of the role of the sulfur vacancies mainly embodied in improving the DOS of SS and reducing the transfer resistance of holes to the water, instead of enhancing the charge recombination. The DOS of SS is further derived from the *C*_trap_ on the basis of the following relationship: *N*_ss_(*E*) = *C*_trap_(*E*)/*q*, where *N*_ss_(*E*) is the DOS of SS as a function of potential, and *q* is the elementary charge^44^. The SS energy distribution follows a Gaussian curve located below the photocurrent onset with its Fermi level pinned at SS (Fig. 4b). The restrained SS distribution and the positive shift of the DOS center of the *V*_s_-CIS-500 photoanode are the thermodynamically fundamental reasons for the larger *V*_ph_ and also account for its high photocurrent density. Besides, the charge carrier density in the space charge region for *V*_s_-CIS-500 (9.94 × 10^20^ cm^−3^) is significantly increased compared to that of CdIn_2_S_4_ (1.02 × 10^19^ cm^−3^), as derived from the Mott–Schottky plots (Fig. 4c, Eq. (9)), which evidences the boosted charge transfer, also in accordance with earlier the DOS calculation for *V*_s_-CIS-500 with S 3*p* shallow trap states to accumulate more charge carriers (Fig. 1g). Notably, a negative shift of the flat band potential for the *V*_s_-CIS-500 photoanode is observed, which illustrates the sharper band bending between *V*_s_-CIS-500 photoanode and electrolyte. Here, the sulfur vacancy with a 2+ charge state has the lowest formation energy according to the DFT calculation. Therefore, the surface sulfur vacancy becomes positively charged ${\mathop{\rm{S}}\nolimits} _{\mathrm{{{vac}}}}^{2 + }$ and donates free carriers to the conduction band of CdIn_2_S_4_ as in other refs. ^45,46^, resulting in the significant increase of the charge carrier density at the surface region. Recent research illustrates that increasing the carrier density provides an effective method to shorten the depletion width (*W*_d_) and enhance the band bending for hole collection^23^. The *W*_d_ of CdIn_2_S_4_ photoanode dramatically decreases from 8.5 to 0.9 nm after introducing surface sulfur vacancies at 1.23 V vs. RHE (Eq. (10)), which indicates a sharper band bending at the *V*_s_-CIS-500/electrolyte interface, resulting in the acceleration of hole drift from depletion region to surface and the suppression of the charge recombination. Therefore, the shortening of the depletion width and enhanced charge separation in *V*_s_-CIS-500 photoanode after introducing surface sulfur vacancies is responsible for the improved photocurrent.

To directly grasp the behavior of photoinduced charge carriers across the Helmholtz layer, we employ intensity-modulated photocurrent spectroscopy (IMPS) to evaluate the pseudo-first-order rate constants of hole transfer (*k*_tran_) and surface recombination (*k*_rec_)^47^. As depicted in Fig. 4d, both the recombination (upper quadrant semicircle) and RC attenuation (lower quadrant semicircle) are quite different between CdIn_2_S_4_ and *V*_s_-CIS-500 photoanodes. The average photoinduced electron transfer time (*τ*_d_) estimated from the frequency at the minimum imaginary part (Eq. (11)) reveals that the *τ*_d_ for the *V*_s_-CIS-500 photoanode is much shorter compared to that of CdIn_2_S_4_ (Supplementary Fig. 18), suggesting its superior charge transfer rate. Moreover, the IMPS spectra at different applied bias are further measured to deduce the values of *k*_tran_ and *k*_rec_ (Supplementary Fig. 19), and the *V*_s_-CIS-500 photoanode manifests an increase in *k*_tran_ and a decrease in *k*_rec_ (Eqs. (12)–(13)) compared to the CdIn_2_S_4_ photoanode (Fig. 4e, f), which are the quantitative evidence for the enhanced hole transfer rate and suppressed surface recombination rate across the Helmholtz layer, and consequently accounts for improved photocurrent density in kinetics.

### DFT calculations

**Fig. 5:**
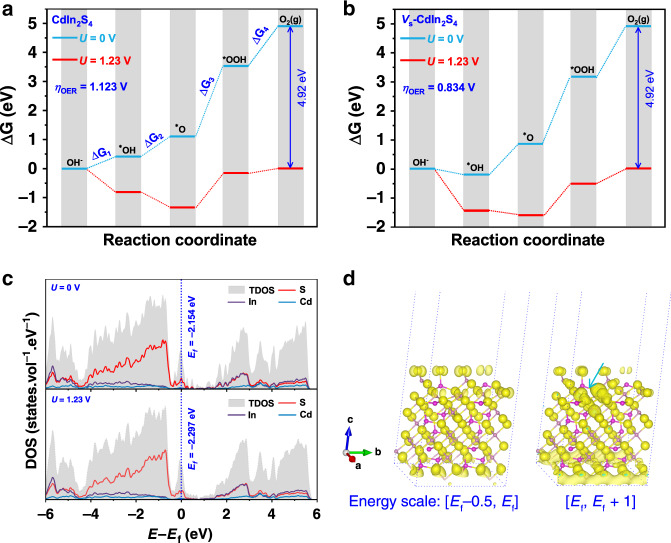
OER mechanism for the CdIn_2_S_4_ photoanode. **a** Free energies of OER steps for CdIn_2_S_4_ ($0\overline 1 1$). **b** Free energies of OER steps for *V*_s_-CdIn_2_S_4_ ($0\overline 1 1$). **c** Comparison of density of states for *V*_s_-CdIn_2_S_4_ ($0\overline 1 1$) without applied bias and with an applied bias of 1.23 V. **d** Photoexcited charge-transition route change for *V*_s_-CdIn_2_S_4_ ($0\overline 1 1$) from HOMO states (in the range of 0–0.5 eV below the Fermi level) to LUMO states (in the range of 0–1 eV above the Fermi level) with an applied bias of 1.23 V (isosurface set at 0.004e Bohr^−3^). The charge difference density is calculated by Δ*ρ* = *ρ*_1.23V_−*ρ*_0V_, and the yellow region represents electron accumulation.

The effect of surface sulfur vacancies on the interfacial OER process was investigated using DFT calculations to elucidate the potential-determining step. Considering that the hole scavengers, that is, H_2_O molecules in the neutral electrolyte must initially absorb on the active site of CdIn_2_S_4_ and *V*_s_-CdIn_2_S_4_ photoanodes to accomplish the following OER steps, the adsorption energy of H_2_O molecules is calculated in consequence. The adsorption energies are determined to be −1.251, and −0.425 eV for CdIn_2_S_4_ and *V*_s_-CdIn_2_S_4_ (Supplementary Note 3), respectively, which indicates decomposition of H_2_O molecules to generate OER intermediates on *V*_s_-CdIn_2_S_4_ is more favorable according to the Sabatier principle^48^. Furthermore, the evolution of three intermediates, OH*, O*, and OOH* involved in the OER processes is further calculated in terms of Gibbs free energy. Interestingly, the stable absorbing geometries of intermediates for *V*_s_-CdIn_2_S_4_ reveal that all the terminal O atoms coordinate with adjacent three In atoms, which is just the location of sulfur vacancy (Supplementary Fig. 26). It confirms the earlier difference charge density calculation result, which manifests the charge accumulation on the adjacent Cd and In atoms (Fig. 1h) after introducing surface sulfur vacancies, beneficial to chemisorption of the OER intermediates. Moreover, the largest Gibbs free energy difference (Fig. 5a, Δ*G*_3_ = 2.418 V, corresponding to *η*_OER_ = 1.123 V) for pristine CdIn_2_S_4_ occurs in the process of formation of OOH* from O*, which illustrates that the strong adsorption of OOH* on CdIn_2_S_4_ is the potential-determining step in the OER process. Besides, despite the unchanged potential-determining step in *V*_s_-CdIn_2_S_4_ (Fig. 5b), the Gibbs free energy is decreased to 2.064 V, resulting in *η*_OER_ remarkably reducing to 0.834 V. The change for the theoretical *η*_OER_ agrees with the experimental overpotentials determined by *J*–*V* curves without illumination (Supplementary Fig. 20), which illustrates that the surface sulfur vacancies reduce the OER overpotential by lowing the formation of OOH*. Notably, the negative Gibbs free energy for the formation of OH* in *V*_s_-CdIn_2_S_4_ without applied bias illustrates that the formation of OH* is a thermodynamically favorable process, indicative of the location of surface sulfur vacancies easily occupied by OH*, which prevents the possible self-oxidation due to the high oxidation potential of photoinduced holes and ensures the stability of this sulfide photoanode.

Given the implementation of applied bias in practical PEC tests, the band structure of *V*_s_-CdIn_2_S_4_ may be changed under applied bias, reflected in the shifts of Fermi level and band offsets in the interfacial region^49,50^. Therefore, the effect of applied bias on the band structure of *V*_s_-CdIn_2_S_4_ is also taken into account. As depicted in Fig. 5c, the Fermi level, and bandgap for *V*_s_-CdIn_2_S_4_ reveal little changes after imposing a bias of 1.23 V, which demonstrates little influence of applied bias on the intrinsic band structure of *V*_s_-CdIn_2_S_4_, further illustrative of the main reason for the improved photocurrent due to the trap states generated by sulfur vacancies. To further unravel the effect of trap states on the charge-transfer routes under the applied bias, photoexcited charge density transition from the sulfur vacancy-induced hybrid highest occupied molecular orbital (HOMO) states to the lowest unoccupied molecular orbital (LUMO) are investigated. As revealed in Fig. 5d, the charge densities of the hybrid HOMO states are nearly unchanged while the charge densities of the hybrid LUMO states are obviously increased in the adjacent In and Cd atoms of sulfur vacancies as indicated by the arrow, illustrative of more charge density generated in the energy range of Fermi level to the LUMO states to facilitate the charge separation and transfer, which is considered to be the reason for the enhanced photocurrent density with the increase of applied bias for the *V*_s_-CIS-500 photoanode.

## Discussion

In summary, we have developed a highly active, deficient ternary sulfide (CdIn_2_S_4_) photoanode for PEC water splitting. The surface sulfur vacancies in CdIn_2_S_4_ are mainly responsible for the improved photocurrent. The effects of surface sulfur vacancies are thoroughly investigated by theoretical calculations and experimental characterizations. The theoretical calculations suggest that introduced surface sulfur vacancies bring about charge accumulation on the adjacent In and Cd atoms, which become the active sites for OER intermediates, resulting in the decreased potential in the rate-determining step. The optical characterizations demonstrate the dominant effects of the surface sulfur vacancies on charge separation and injection efficiencies, indicative of the shallow trap states introduced by sulfur vacancies facilitating the charge separation and transfer. The characterizations of kinetics about charge transfer and recombination further confirmed the improved hole transfer rate and suppressed surface charge recombination in the SS, which are the fundamental reasons for the enhanced photocurrent. Benefiting from the surface sulfur vacancies, the CdIn_2_S_4_ photoanode reveals a photocurrent density of 5.73 mA cm^−2^ at 1.23 V vs. RHE and an ABPE value of 2.49% at 0.477 V vs. RHE, which make it a promising candidate for PEC water splitting.

## Methods

### Synthesis of the CdIn_2_S_4_ photoanode

The fluorine-doped tin oxide conductive glasses (FTO, 10 Ω sq^−1^) were pretreated by successive sonication in acetone, ethanol, and distilled water. In a typical procedure, stoichiometric amounts of Cd(NO_3_)_2_·4H_2_O (0.6 mmol), In(NO_3_)_3_·5H_2_O (1.2 mmol), and excessive amounts of thioacetamide (CH_3_CSNH_2_) (9.6 mmol) were dissolved in 35 mL of distilled water (Milli-Q) and then transferred into a 50 mL Teflon-lined stainless autoclave. Subsequently, the pretreated FTO was placed at an angle against the wall of the Teflon-liner with the conductive side facing down. The autoclave was sealed and maintained at 160 °C for 10 h, followed by cooling naturally to room temperature. Finally, the FTO substrate deposited with CdIn_2_S_4_ film was rinsed with distilled water and dried in air at room temperature.

### Synthesis of the *V*_s_-CIS photoanode

In a typical procedure, the as-synthesized CdIn_2_S_4_ photoanode was annealed for 2 h under H_2_/Ar-mixed gas flux (1:10 vol/vol, 100 mL min^−1^) atmosphere at a temperature of 350–550 °C with a heating rate of 5 °C min^−1^. After cooling naturally to room temperature, the *V*_s_-CIS photoanode was obtained and denoted as *V*_s_-CIS-*T* (*T* refers to the annealing temperature).

### Characterizations

PXRD patterns were collected on a Bruker AXS D8 diffractometer in the Bragg–Brentano mode, using Cu Kα radiation (1.5418 Å). The morphology and microstructure of the samples were investigated by transmission electron microscopy (TEM, JEOL JEM-1011) and field-emission scanning electron microscopy (FE-SEM, SU8010). The lattice structures were observed by high-resolution transmission electron microscopy (HRTEM, JEOL JEM-2100) and spherical aberration-corrected TEM (Titan Cubed Themis G2 300). The XPS and valence-band XPS (VB-XPS) were recorded on a Thermo scientific ESCALAB 250Xi with 30.0 eV pass energy and an Al Kα line excitation source, using C 1*s* (binding energy of 284.8 eV) as a reference. The UPS was conducted with a monochromatic He light source (21.22 eV), and a bias of 8 eV was applied to observe the secondary electron cutoff edge. The optical properties of samples were investigated by an ultraviolet–visible spectrophotometer (Cary 100, Agilent Technologies). The PL spectra (excited by 370 nm light illumination) were measured using a fluorescence spectrophotometer (G9800A, Agilent Technologies). The TRPL spectroscopy under the excitation of 377.8 nm laser was performed on a fluorescence lifetime spectrophotometer (FLS920, Edinburgh). The ESR spectra were recorded on a spectrometer (JES-X320, JEOL) at 300 K and 9165.365 MHz. SPV spectroscopy was carried out on the basis of a lock-in amplifier to study the separation characteristics of the photogenerated charges by the spectrometer (CEL-SPS1000, Ceaulight Technology Co. Ltd., China).

### PEC property measurements

PEC measurements were carried out on an electrochemical workstation (CHI 760e, CH Instruments Inc., China) at room temperature with a conventional three-electrode cell. The prepared photoanode with an exposed area of 1 × 1 cm^2^ was employed as the working electrode, while the saturated Ag/AgCl electrode and Pt foil were used as the reference electrode and counter electrode, respectively. A 300 W xenon arc lamp (CEL-PE300L-3A) with a filter (AM 1.5 G, Ceaulight Technology Co. Ltd., China) was employed to simulate solar illumination with about 1 Sun power. The back-side illumination through the FTO side was adopted for all the PEC tests. The photo-oxidation of sulfite performance was measured in a 0.35 M Na_2_SO_3_ and 0.25 M Na_2_S-mixed solution (pH = 12.5), while the photo-oxidation of water performance was measured in a 0.5 M Na_2_SO_4_ solution (pH = 7). To study the kinetics of charge transfer and recombination, 0.5 M Na_2_SO_4_ solution (pH = 7) was used as the electrolyte. The amounts of evolved H_2_ and O_2_ were determined at regular time intervals using GC (3420A, Beifen-Ruili Co. Ltd., China) with a thermal conductivity detector and a 5 Å molecular sieve column.

During the evaluation of the PEC performance of CdIn_2_S_4_ and *V*_s_-CIS photoanodes, all measured potentials were converted to reversible hydrogen electrode (RHE) using the Nernst equation below.

1 $$E_{{\mathrm{RHE}}} = E_{{\mathrm{Ag/AgCl}}} + E_{{\mathrm{Ag/AgCl}}}\left( {{\mathrm{reference}}} \right) + 0.0591\,{\kern 1pt} {\mathrm{V}} \times {\mathrm{pH}}$$

(*E*_Ag/AgCl_(reference) = 0.1976 V vs. NHE at 25 °C)

The ABPE was calculated from the *J–V* curve using the equation^51^:

2 $${\mathrm{{ABPE}}} = \left[ {\frac{{{\it{j}}_{\mathrm{{{ph}}}}\left( {{\mathrm{{mA}}}{\kern 1pt} \,{\mathrm{{cm}}}^{ - 2}} \right) \times \left( {1.23 - {\it{V}}_{{\mathrm{{bias}}}}} \right)\left( {\mathrm{{V}}} \right)}}{{{\it{P}}_{{\mathrm{{total}}}}\left( {{\mathrm{{mW}}}\,{\kern 1pt} {\mathrm{{cm}}}^{ - 2}} \right)}}} \right]_{\mathrm{{{AM1.5G}}}}$$

where *j*_ph_ is the photocurrent density obtained under an applied bias (*V*_bias_), and *P*_total_ is the incident illumination power density.

The IPCE at different wavelengths was measured at 0.6 V vs. RHE using monochromatic light illumination from a 300 W Xe arc lamp equipped with a monochromator (CEL-QPCE3000, Ceaulight Technology Co. Ltd., China). The IPCE values were determined using the equation^51^:

3 $${\mathrm{{IPCE}}}\left( {\it{\lambda }} \right) = \frac{{{\it{j}}_{\mathrm{{{ph}}}}\left( {{\mathrm{{mA}}}\,{\kern 1pt} {\mathrm{{cm}}}^{ - 2}} \right) \times 1239.8\left( {{\mathrm{{V}}} \times {\mathrm{{nm}}}} \right)}}{{{\it{P}}_{{\mathrm{{mono}}}}\left( {{\mathrm{{mW}}}\,{\kern 1pt} {\mathrm{{cm}}}^{ - 2}} \right) \times {\it{\lambda }}\left( {\mathrm{{{nm}}}} \right)}}$$

where 1239.8 V nm represents a multiplication of *h* (Planck’s constant) and *c* (the speed of light), *λ* is the incident light wavelength (nm), and *P*_mono_ is the monochromated illumination power intensity.

### Supplemented equations for evaluating photocurrent density

4 $${\it{J}}_{{\mathrm{ph}}} = {\it{J}}_{{\mathrm{{abs}}}} \times {\it{\eta }}_{{\mathrm{{seperation}}}} \times {\it{\eta }}_{{\mathrm{{injection}}}}$$

5 $${\it{J}}_{{\mathrm{{abs}}}}{\it{ = }}\frac{{\it{q}}}{{{\it{hc}}}}\mathop {\int}\limits_{\it{\lambda }} {{\it{\lambda }}\phi _\lambda {\it{\eta }}_{{\mathrm{{abs}}}}} {{\mathrm{{d}}}\lambda }$$

6 $${\it{\eta }}_{{\mathrm{seperation}}} = {\it{J}}_{{\mathrm{ph}}}^{{{{\rm{Na}}_2{\rm{SO}}_3}}/{\mathrm{Na}}_{\mathrm{2}}{\mathrm{S}}}/{\it{J}}_{{\mathrm{abs}}}$$

7 $${\it{\eta }}_{{\mathrm{injection}}} = {\it{J}}_{{\mathrm{ph}}}^{{\mathrm{Na}}_{\mathrm{2}}{\mathrm{SO}}_{\mathrm{4}}}/{\it{J}}_{{\mathrm{ph}}}^{{{{\rm{Na}}_2{\rm{SO}}_3}}/{\mathrm{Na}}_{\mathrm{2}}{\mathrm{S}}}$$

8 $${\it{\eta }}_{{\mathrm{abs}}} = \left( {1 - 10^{ - {\it{A}}}} \right) \times 100\%$$

The ${\it{J}}_{{\mathrm{ph}}}^{{{{\rm{Na}}_2{\rm{SO}}_3/{\rm{Na}}_2{\rm{S}}}}}$ is the photocurrent density measured in 0.35 M Na_2_SO_3_ and 0.25 M Na_2_S mixed electrolyte, which serves as hole scavengers and ensures the hole injection rate approaching 100%, and ${\it{J}}_{{\mathrm{ph}}}^{{{{\rm{Na}}_2{\rm{SO}}_4}}}$ is the photocurrent densities measured in 0.5 M Na_2_SO_4_. The *J*_abs_ is the photon adsorption rate expressed as the photocurrent density, and *q* is the charge of an electron, *h* is the Plank constant, *c* is the light speed, *ϕ*_*λ*_ is the photon flux of the AM 1.5 G solar spectrum, and *η*_abs_ is the light absorption efficiency^52^.

### Measurement of Mott–Schottky plots

The charge carrier density in the space charge region was measured in a 0.5 M Na_2_SO_4_ solution at a frequency of 1 kHz in the dark, and calculated according to the Mott–Schottky equation^53^

9 $$\frac{1}{{{\mathrm{C}}^2}} = \frac{2}{{{\it{\varepsilon \varepsilon }}_{\it{0}}{\it{A}}^2{\it{qN}}_{\mathrm{{D}}}}} \times \left( {{\it{V}} - {\it{V}}_{{\mathrm{fb}}} - \frac{{{\it{k}}_{\mathrm{B}}{\it{T}}}}{q}} \right)$$

where *C* is the space-charge capacitance, *V* (*V* vs. RHE) is the applied voltage, *V*_fb_ (*V* vs. RHE) is the flat-band potential, *N*_D_ is the charge carrier density, *ε* is the dielectric constant of the semiconductor (taken as 6.6 for the CdIn_2_S_4_^54^), *ε*_0_ is the vacuum permittivity (8.854 × 10^−12^ C V^−1^ m^−1^), *k*_B_ is Boltzmann’s constant (1.381 × 10^−23^ J K^−1^), *q* is the electronic charge (1.602 × 10^−19^ C), and *T* is the absolute temperature.

The thickness of the depletion width (*W*_d_) can be calculated as^55^

10 $$W_{\mathop{\rm{d}}\nolimits} = \left[ {\frac{{2\varepsilon _o\varepsilon \left( {V - V_{{\mathop{\rm{fb}}\nolimits} }} \right)}}{{qN_{\mathrm{{D}}}}}} \right]^{1/2}$$

### IMPS and PEIS measurements

The IMPS and PEC impedance spectroscopy (PEIS) measurements were conducted on an electrochemical workstation (CIMPS-Pro, Zahner Co.) in 0.5 M Na_2_SO_4_ solution with a three-electrode configuration at different bias potentials.

For the IMPS measurement, modulated illumination was provided by a high-intensity light-emitting diode (LED: LSW-2) controlled by a LED driver (PP211) that allowed the superimposition of sinusoidal modulation (~10%) on a dc illumination level. The wavelength of light was 430–720 nm in the visible light region with an average intensity of 100 mW cm^−2^. The modulation amplitude of the lamp voltage was 100 mV. The photocurrent as a function of frequency (from 0.1 to 10 kHz) after the light turned on was recorded.

In the typical IMPS response, the average photogenerated electron transfer time (*τ*_d_) can be estimated from the frequency at the minimum imaginary part^56^

11 $$\tau _{\mathop{\rm{d}}\nolimits} = \frac{1}{{2\pi f_{{\mathrm{min}}}}}$$

The frequency at the maximum imaginary part corresponds to the sum of the charge transfer (*k*_tran_) and recombination (*k*_rec_) rate constants as expressed^56^

12 $$k_{{\mathrm{tran}}} + k_{{\mathrm{rec}}} = 2\pi f_{\max }$$

The hole transfer efficiency (*η*_tran_) at the semiconductor/electrolyte interface can be determined by the ratio of the steady-state photocurrent (*j*_ss_) to the instantaneous photocurrent (*j*_hole_). Assuming that both hole transfer and recombination are pseudo-first-order in the surface hole concentration, the hole transfer efficiency can also be expressed by the ratio of *k*_rec_ and *k*_tran_^57^

13 $$\eta _{{\mathrm{tran}}} = \frac{{j_{{\mathrm{ss}}}}}{{j_{{\mathrm{hole}}}}} = \frac{{k_{{\mathrm{tran}}}}}{{k_{{\mathrm{tran}}} + k_{{\mathrm{rec}}}}}$$

The PEIS measurements were carried out in a frequency range of 0.1 Hz to 100 kHz with an amplitude of 5 mV under constant light (430–720 nm, 100 mW cm^−2^) illumination. The Randles equivalent circuit was used to analyze the impedance data using Zview software (Scribner Associates).

### PALS measurement

PALS measurements were carried out with a fast–fast coincidence system with a time resolution of 190 ps full width at half-maximum (FWHM) for the *γ*-rays from a ^60^Co source selected under the experimental conditions. The sample powder was pressed into a disk (diameter: 10.0 mm, thickness: 1.0 mm). A 4 × 10^5^ Bq source of ^22^Na was sandwiched between two identical sample disks. The positron lifetime spectrum containing 2 × 10^6^ counts were analyzed by the computer program Limetime9.0 to decompose several lifetime components.

### DFT calculations for surface energy

The first-principle calculations corrected by on-site Coulomb interaction were carried out with the Vienna ab initio simulation package (VASP)^58,59^. The interaction between ions and valence electrons was described using the projector-augmented wave (PAW) potentials, and the exchange-correction function was treated using the generalized gradient approximation (GGA) in the Perdew–Burke–Ernzerhof for solid (PBEsol) form^60^. Interactions between the valence electrons and the ion core are represented in 4*d*^10^5*s*^2^, 4*d*^10^5*s*^2^5*p*^1^, and 3*s*^2^3*p*^4^ orbitals for Cd, In and S, respectively. The wave functions were expanded in a plane wave basis with an energy cutoff of 500 eV, which was high enough to ensure that no Pulay stresses occur within the cell during geometry relaxation. The effective *U–J* values of 2.1 and 1.9 were employed to account for the strong on-site Coulomb interaction of Cd and In atoms^61^, respectively. The Brillouin zone was sampled by a Γ-centered method, and a K-points resolved value of 0.02 was employed for all the geometries optimization, which was set to 0.01 for the density of states calculations. The Fermi level was slightly broadened using Fermi–Dirac smearing of 50 meV. For all the calculations, the convergence criteria for the electronic and ionic relaxation are 10^−5^ eV and 0.02 eV/Å, respectively.

Based on the BFDH theory and the experimental morphology of CdIn_2_S_4_ (Fig. 1e), we constructed the geometry of CdIn_2_S_4_, which was consisted of {100}, {110}, and {$0\overline 1 1$} family of crystal planes. Given the effect of truncated atoms on the surface energy, (100), (110), and ($0\overline 1 1$) crystal planes with variable truncated atoms were taken into account. The symmetrical slab models with a vacuum thickness of 30 Å were adopted to simulate the geometries of possible exposed planes (Supplementary Fig. 21), and the surface energies in their possible chemical potentials were calculated according to the following equation:

14 $$\gamma {\mathrm{ = }}\frac{1}{{2A}}\left( {E_{\mathrm{s}}^{{\mathrm{{relax}}}} - {n}_{{\mathrm{Cd}}}\mu _{{\mathrm{Cd}}}{ - n}_{{\mathrm{In}}}\mu _{{\mathrm{In}}}{ - n}_{\mathrm{S}}\mu _{\mathrm{S}}} \right)$$

where *A* is the surface area,$E_{\mathrm{{s}}}^{{\mathrm{{relax}}}}$is the total energy of the relaxed slab, and *n*_Cd_*, n*_In_, and *n*_S_ are the numbers of Cd, In, and S involved in the slab model, *μ*_Cd_*, μ*_In_, and *μ*_S_ are the atomic chemical potential of Cd, In, and S atoms, respectively. The factor of 1/2 corresponded to two equivalent surfaces in the slab models (see details in Supplementary Fig. S22 and Supplementary Table 3).

### DFT calculations for the sulfur vacancy

To illustrate the most feasible plane for the formation of surface sulfur vacancy, slabs models with vacuum thickness of 15 Å were constructed, as shown in Supplementary Fig. 23. To minimize the effect of spurious electrostatic interactions in charged defect calculations due to the periodic cell approximation, a 2 × 2 periodic surfaces were employed to ensure the distance of the sulfur vacancies in adjacent cells over 10 Å. The bottom atoms were saturated with H and pseudo-potential 0.5H to remove the dangling bonds. The possibility of production of the surface sulfur vacancy in (100), (110), and ($0\overline 1 1$) crystal planes was evaluated by the formation energy which is estimated as^62^,

15 $$\Delta H_{\mathop{\rm{f}}\nolimits} \left( {X^q} \right) = E_{{\mathrm{{tot}}}}\left( {{\mathop{\rm{X}}\nolimits} ^q} \right) - E_{{\mathop{\rm{tot}}\nolimits} }\left( {{\mathop{\rm{bulk}}\nolimits} } \right) - \mathop {\sum}\limits_{i} {n_i\mu _i + q\left( {E_{\mathrm{{v}}} + E_{\mathrm{{F}}}} \right)}$$

where *E*_tot_(*X*^*q*^) and *E*_tot_(bulk) are the total energies of the defect *X* with charge *q* and bulk, respectively. The *n*_*i*_ is the number of atoms being added to (*n*_*i*_ > 0) and/or removed from (*n*_*i*_ < 0) in the perfect-crystal supercell, and *μ*_*i*_ is the atomic chemical potential. *E*_VBM_ is the energy of the valence-band maximum (VBM), and *E*_F_ is the Fermi level measured from the VBM, varying in the range of the bandgap *E*_g_ (see details in Supplementary Fig. 24 and Supplementary Fig. 25).

### DFT calculations for surface OER mechanism

The slab models of ($0\overline 1 1$) planes with In and S as truncated atoms were constructed to elucidate the potential-determining step in surface OER process by calculating the evolution of OH*, O*, and OOH* intermediates on CdIn_2_S_4_ and deficient CdIn_2_S_4_. As shown in Supplementary Fig. 26, the slab model consisted of eight atomic layers and a vacuum thickness of 15 Å. Similarly, the bottom atoms were saturated with H and pseudo-potential 0.5H to remove the dangling bonds (see details in Supplementary Note 3).

## Supplementary information

supplementary information

Peer Review File

## Data Availability

The data that support the findings of this study are available from the corresponding authors upon reasonable request.

## References

[1] Roger I, Shipman MA, Symes MD (2017). Earth-abundant catalysts for electrochemical and photoelectrochemical water splitting. Nat. Rev. Chem..

[2] Montoya JH (2017). Materials for solar fuels and chemicals. Nat. Mater..

[3] Jiang C, Moniz SJ, Wang A, Zhang T, Tang J (2017). Photoelectrochemical devices for solar water splitting–materials and challenges. Chem. Soc. Rev..

[4] Yao T, An X, Han H, Chen JQ, Li C (2018). Photoelectrocatalytic materials for solar water splitting. Adv. Energy Mater..

[5] Shen S, Lindley SA, Chen X, Zhang JZ (2016). Hematite heterostructures for photoelectrochemical water splitting: rational materials design and charge carrier dynamics. Energy Environ. Sci..

[6] Kuang Y (2016). A front‐illuminated nanostructured transparent BiVO_4_ photoanode for >2% efficient water splitting. Adv. Energy Mater..

[7] Liu G (2016). Enabling an integrated tantalum nitride photoanode to approach the theoretical photocurrent limit for solar water splitting. Energy Environ. Sci..

[8] Kim ES (2013). Fabrication of CaFe_2_O_4_/TaON heterojunction photoanode for photoelectrochemical water oxidation. J. Am. Chem. Soc..

[9] Ning F (2016). TiO_2_/graphene/NiFe-layered double hydroxide nanorod array photoanodes for efficient photoelectrochemical water splitting. Energy Environ. Sci..

[10] Sivula K, Van De Krol R (2016). Semiconducting materials for photoelectrochemical energy conversion. Nat. Rev. Mater..

[11] Chandrasekaran S (2019). Recent advances in metal sulfides: from controlled fabrication to electrocatalytic, photocatalytic and photoelectrochemical water splitting and beyond. Chem. Soc. Rev..

[12] Guo Q (2019). Efficient and selective CO_2_ reduction integrated with organic synthesis by solar energy. Chem.

[13] Kuo T-R (2018). Extended visible to near-infrared harvesting of earth-abundant FeS_2_–TiO_2_ heterostructures for highly active photocatalytic hydrogen evolution. Green Chem..

[14] Wang Y (2017). Three-dimensional WO_3_ nanoplate/Bi_2_S_3_ nanorod heterojunction as a highly efficient photoanode for improved photoelectrochemical water splitting. ACS Appl. Mater. Int..

[15] Zhou M (2019). Hybrid 0D/2D edamame shaped ZnIn_2_S_4_ photoanode modified by Co-Pi and Pt for charge management towards efficient photoelectrochemical water splitting. Appl. Catal. B–Environ..

[16] Ran L, Yin L (2019). Ternary Hierarchical Cu_7_S_4_/TiO_2_/CoCr‐LDH heterostructured nanorod arrays with multiphase reaction interfaces for more efficient photoelectrochemical water splitting. Adv. Mater. Int..

[17] Song J-P, Yin P-F, Mao J, Qiao S-Z, Du X-W (2017). Catalytically active and chemically inert CdIn_2_S_4_ coating on a CdS photoanode for efficient and stable water splitting. Nanoscale.

[18] Sinsermsuksakul P (2014). Overcoming efficiency limitations of SnS‐based solar cells. Adv. Energy Mater..

[19] Meng L (2019). Doping-induced amorphization, vacancy, and gradient energy band in SnS_2_ nanosheet arrays for improved photoelectrochemical water splitting. Angew. Chem. Int. Ed..

[20] Giri B (2019). Balancing light absorption and charge transport in vertical SnS_2_ nanoflake photoanodes with stepped layers and large intrinsic mobility. Adv. Energy Mater..

[21] Fu Y (2018). Phase-modulated band alignment in CdS nanorod/SnS_x_ nanosheet hierarchical heterojunctions toward efficient water splitting. Adv. Funct. Mater..

[22] Tian Z (2020). Enhanced charge carrier lifetime of TiS_3_ photoanode by introduction of $${\mathrm{S}}_{2}^{2-} $$ vacancies for efficient photoelectrochemical hydrogen evolution. Adv. Funct. Mater..

[23] Tian Z (2019). Novel Black $$BiVO_{4}/TiO_{2-x} $$ photoanode with enhanced photon absorption and charge separation for efficient and stable solar water splitting. Adv. Energy Mater..

[24] Li X (2019). Selective visible-light-driven photocatalytic CO_2_ reduction to CH_4_ mediated by atomically thin CuIn_5_S_8_ layers. Nat. Energy.

[25] Wang G (2011). Hydrogen-treated TiO_2_ nanowire arrays for photoelectrochemical water splitting. Nano Lett..

[26] Sun X (2019). Enhanced superoxide generation on defective surfaces for selective photo-oxidation. J. Am. Chem. Soc..

[27] Donnay JDH, Harker D (1937). A new law of crystal morphology extending the law of Bravais. Am. Mineral..

[28] Yang X, Du C, Liu R, Xie J, Wang D (2013). Balancing photovoltage generation and charge-transfer enhancement for catalyst-decorated photoelectrochemical water splitting: a case study of the hematite/MnO_*x*_ combination. J. Catal..

[29] Du C (2013). Hematite‐based water splitting with low turn‐on voltages. Angew. Chem. Int. Ed..

[30] Shao M, Ning F, Wei M, Evans DG, Duan X (2014). Hierarchical nanowire arrays based on ZnO core-layered double hydroxide shell for largely enhanced photoelectrochemical water splitting. Adv. Funct. Mater..

[31] Wang H (2019). Interfacial coupling effect on electron transport in hierarchical TaON/Au/ZnCo-LDH photoanode with enhanced photoelectrochemical water oxidation. ACS Appl. Mater. Int..

[32] Chen R (2018). Giant defect-induced effects on nanoscale charge separation in semiconductor photocatalysts. Nano Lett..

[33] Kronik L, Shapira Y (2001). Surface photovoltage spectroscopy of semiconductor structures: at the crossroads of physics, chemistry and electrical engineering. Surf. Interface Anal..

[34] Bae D, Seger B, Vesborg PCK, Hansen O, Chorkendorff I (2017). Strategies for stable water splitting via protected photoelectrodes. Chem. Soc. Rev..

[35] Chen S, Takata T, Domen K (2017). Particulate photocatalysts for overall water splitting. Nat. Rev. Mater..

[36] Ran J, Ma TY, Gao G, Du X-W, Qiao SZ (2015). Porous P-doped graphitic carbon nitride nanosheets for synergistically enhanced visible-light photocatalytic H_2_ production. Energy Environ. Sci..

[37] Dotan H, Sivula K, Grätzel M, Rothschild A, Warren SC (2011). Probing the photoelectrochemical properties of hematite (α-Fe_2_O_3_) electrodes using hydrogen peroxide as a hole scavenger. Energy Environ. Sci..

[38] Kong M (2011). Tuning the relative concentration ratio of bulk defects to surface defects in TiO_2_ nanocrystals leads to high photocatalytic efficiency. J. Am. Chem. Soc..

[39] Li L (2015). Sub-10 nm rutile titanium dioxide nanoparticles for efficient visible-light-driven photocatalytic hydrogen production. Nat. Commun..

[40] Monllor-Satoca D (2015). What do you do, titanium? Insight into the role of titanium oxide as a water oxidation promoter in hematite-based photoanodes. Energy Environ. Sci..

[41] Klahr B, Gimenez S, Fabregat-Santiago F, Hamann T, Bisquert J (2012). Water oxidation at hematite photoelectrodes: the role of surface states. J. Am. Chem. Soc..

[42] Klahr B, Gimenez S, Fabregat-Santiago F, Bisquert J, Hamann TW (2012). Electrochemical and photoelectrochemical investigation of water oxidation with hematite electrodes. Energy Environ. Sci..

[43] Tang P (2017). Enhanced photoelectrochemical water splitting of hematite multilayer nanowire photoanodes by tuning the surface state via bottom-up interfacial engineering. Energy Environ. Sci..

[44] Bisquert J (2003). Chemical capacitance of nanostructured semiconductors: its origin and significance for nanocomposite solar cells. Phys. Chem. Chem. Phys..

[45] Wang S (2018). New BiVO_4_ dual photoanodes with enriched oxygen vacancies for efficient solar-driven water splitting. Adv. Mater..

[46] Feng S (2020). Enriched surface oxygen vacancies of photoanodes by photoetching with enhanced charge separation. Angew. Chem. Int. Ed..

[47] Zachäus C, Abdi FF, Peter LM, Van De Krol R (2017). Photocurrent of BiVO_4_ is limited by surface recombination, not surface catalysis. Chem. Sci..

[48] Medford AJ (2015). From the Sabatier principle to a predictive theory of transition-metal heterogeneous catalysis. J. Catal..

[49] Huang L (2015). Electric-field tunable band offsets in black phosphorus and MoS_2_ van der Waals p–n heterostructure. J. Phys. Chem. Lett..

[50] Yu L, Ruzsinszky A, Perdew JP (2016). Bending two-dimensional materials to control charge localization and Fermi-level shift. Nano Lett..

[51] Chen Z (2010). Accelerating materials development for photoelectrochemical hydrogen production: standards for methods, definitions, and reporting protocols. J. Mater. Res..

[52] Zhong DK, Choi S, Gamelin DR (2011). Near-complete suppression of surface recombination in solar photoelectrolysis by “Co-Pi” catalyst-modified W: BiVO_4_. J. Am. Chem. Soc..

[53] Kim JH (2016). Hetero-type dual photoanodes for unbiased solar water splitting with extended light harvesting. Nat. Commun..

[54] Sawant R, Rajpure K, Bhosale C (2007). Determination of CdIn_2_S_4_ semiconductor parameters by (photo) electrochemical technique. Physica B.

[55] Li Z, Luo W, Zhang M, Feng J, Zou Z (2013). Photoelectrochemical cells for solar hydrogen production: current state of promising photoelectrodes, methods to improve their properties, and outlook. Energy Environ. Sci..

[56] Ponomarev E, Peter L (1995). A generalized theory of intensity modulated photocurrent spectroscopy (IMPS). Electroanal. Chem..

[57] Peter LM, Wong LH, Abdi FF (2017). Revealing the influence of doping and surface treatment on the surface carrier dynamics in hematite nanorod photoanodes. ACS Appl. Mater. Int..

[58] Kresse G, Hafner J (1993). Ab initio molecular dynamics for liquid metals. Phys. Rev. B.

[59] Kresse G, Furthmüller J (1996). Efficiency of ab-initio total energy calculations for metals and semiconductors using a plane-wave basis set. Comp. Mater. Sci..

[60] Perdew JP (2008). Restoring the density-gradient expansion for exchange in solids and surfaces. Phys. Rev. Lett..

[61] Curtarolo S (2012). AFLOW: an automatic framework for high-throughput materials discovery. Comp. Mater. Sci..

[62] Walle CGVd, Neugebauer J (2004). First-principles calculations for defects and impurities: applications to III-nitrides. J. Appl. Phys..

