# Supplementary material for: Highly active deficient ternary sulfide photoanode for photoelectrochemical water splitting
Source: Nat Commun. 2020 Jun 17;11:3078. doi: 10.1038/s41467-020-16800-w (PMC7299993; doi:10.1038/s41467-020-16800-w)
Supplement: Supplementary file 1 — supplementary information [file 41467_2020_16800_MOESM1_ESM.pdf]

Supplementary Information

Highly active deficient ternary sulfide photoanode for photoelectrochemical water splitting

Wang *et al*.

Supplementary Figures

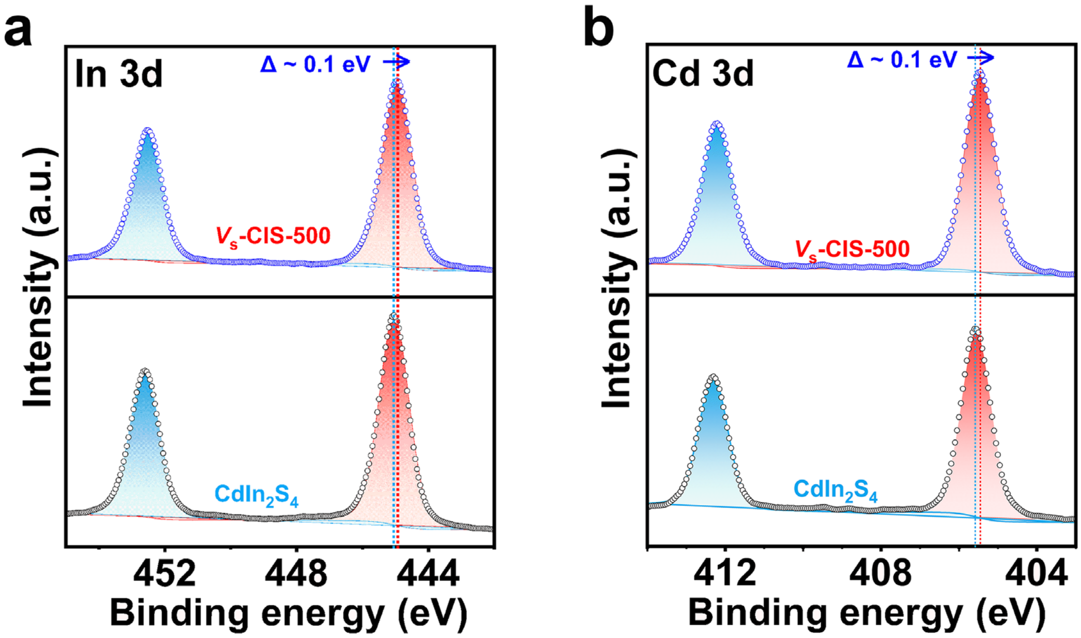

Supplementary Figure 1. XPS spectra for In 3d and Cd 3d. Core-level XPS spectra of (**a**) In 3d and (**b**) Cd 3d for pristine $CdIn_2S_4$ and V_s-CIS-500 samples.

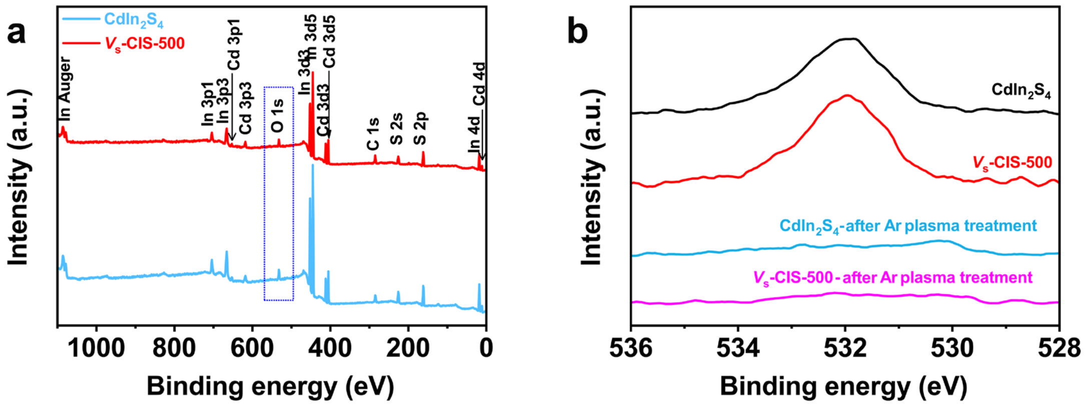

Supplementary Figure 2. **XPS spectra to illustrate the surface hydroxyls**. (**a**) XPS full spectra for $CdIn_2S_4$ and V_s-CIS-500, and (**b**) XPS spectra of O1s for $CdIn_2S_4$ and V_s-CIS-500, and samples after Ar plasma treatment.

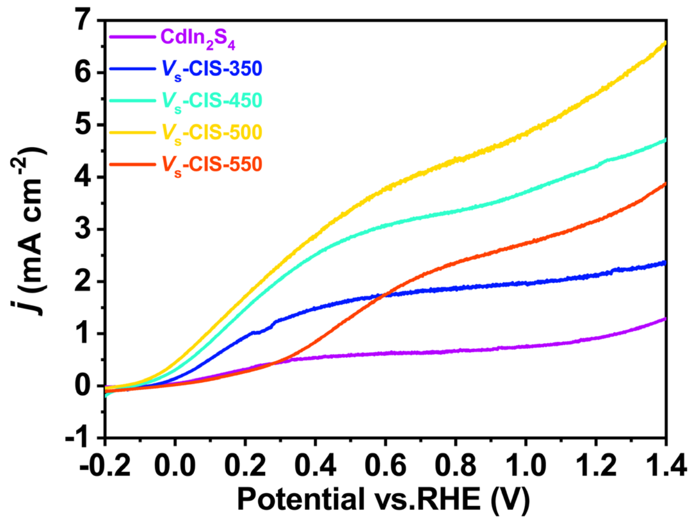

Supplementary Figure 3. J-V curves. Current density-voltage plots for as-prepared products measured in 0.35 M Na_2SO_3 and 0.25 M Na_2S mixed solution (pH = 12.5) under AM 1.5G, 100 mW cm^{-2} illumination with a scan rate of 10 mV s^{-1}.

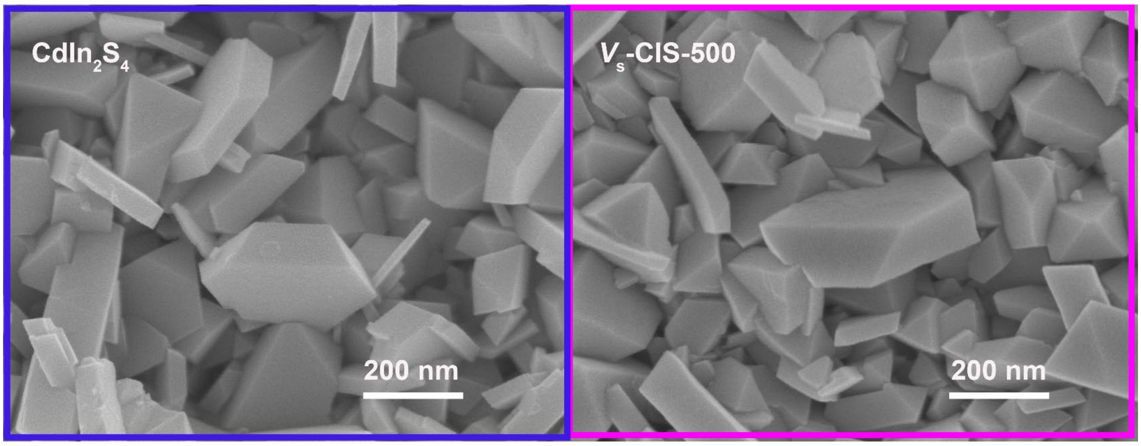

Supplementary Figure 4. Morphology comparison. The SEM images of pristine $CdIn_2S_4$ and V_s-CIS-500 samples.

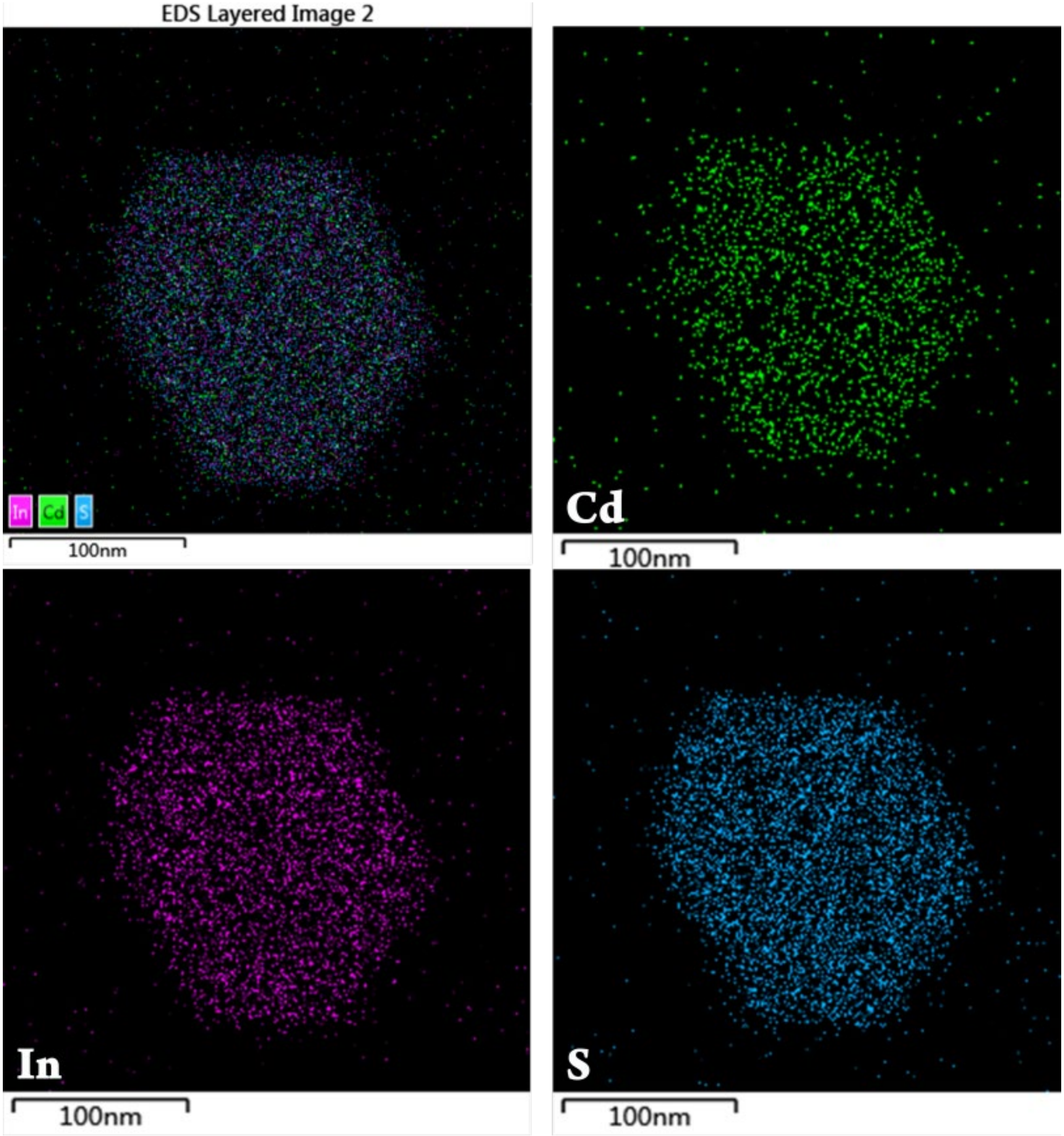

Supplementary Figure 5. The EDS elemental mapping images of V_s-CIS-500.

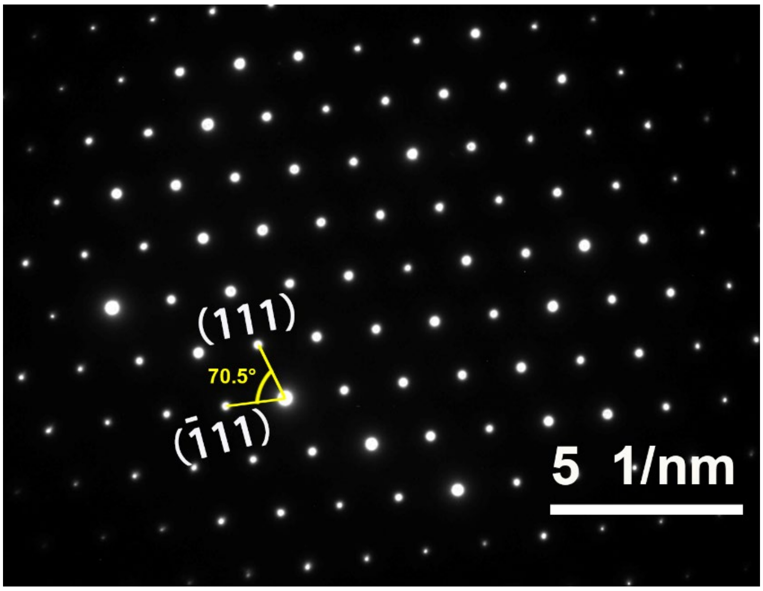

Supplementary Figure 6. The SAED image of the V_s-CIS-500 sample. The crystal lattices of the SAED image reveal lattice points assigned to (111) and $(\bar{1}11)$ to form a dihedral angle of 70.5° in the reciprocal space. Therefore, the exposed plane should be the $(0\bar{1}1)$ crystal plane.

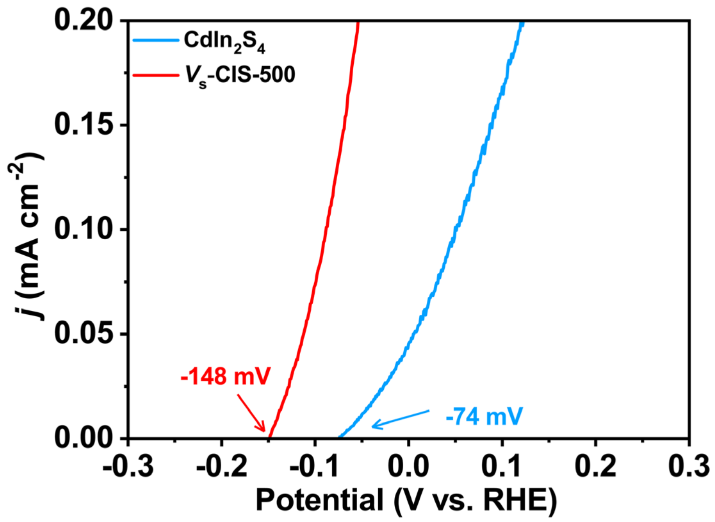

Supplementary Figure 7. Onset potential determination. The onset potentials of the $CdIn_2S_4$ and V_s-CIS-500 photoanodes are determined through the intersection points of the *J*-*V* curves and the dark current curves.

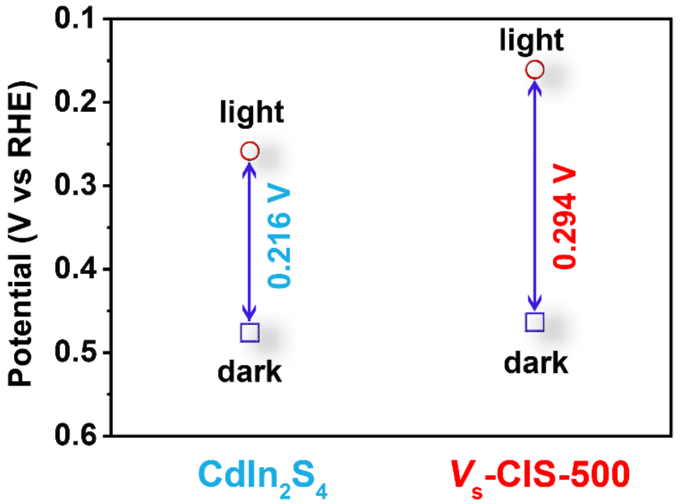

Supplementary Figure 8. The photovoltage positions. Detailed photovoltage positions of $CdIn_2S_4$ and V_s-CIS-500 photoanodes derived from open-circuit voltage decay plots in the dark and under illumination.

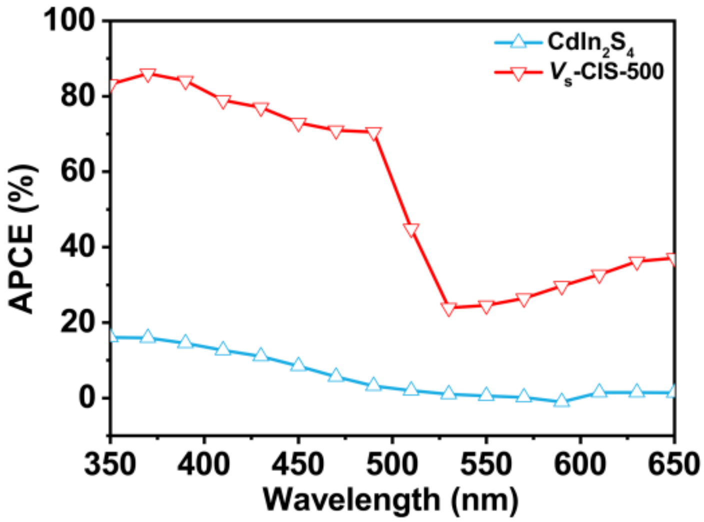

Supplementary Figure 9. APCE values for $CdIn_2S_4$ and V_s-CIS-500 photoanodes.

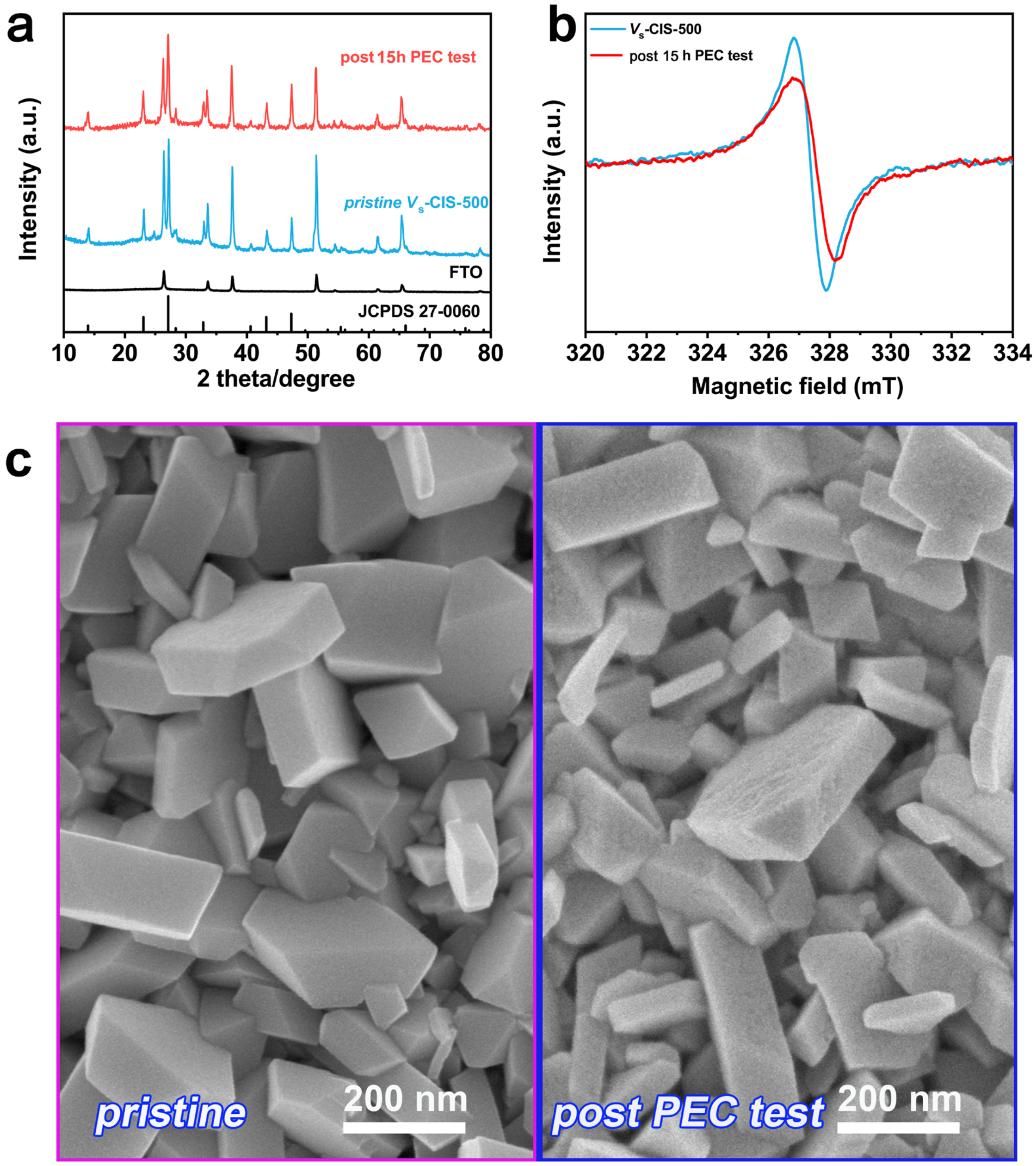

Supplementary Figure 10. Physicochemical characterizations to illustrate the structural stability of the V_s-CIS-500 photoanode. Comparison of (**a**) XRD patterns, (**b**) ESR signals, and (**c**) SEM images between the pristine V_s-CIS-500 photoanode and that after long-term J-t test.

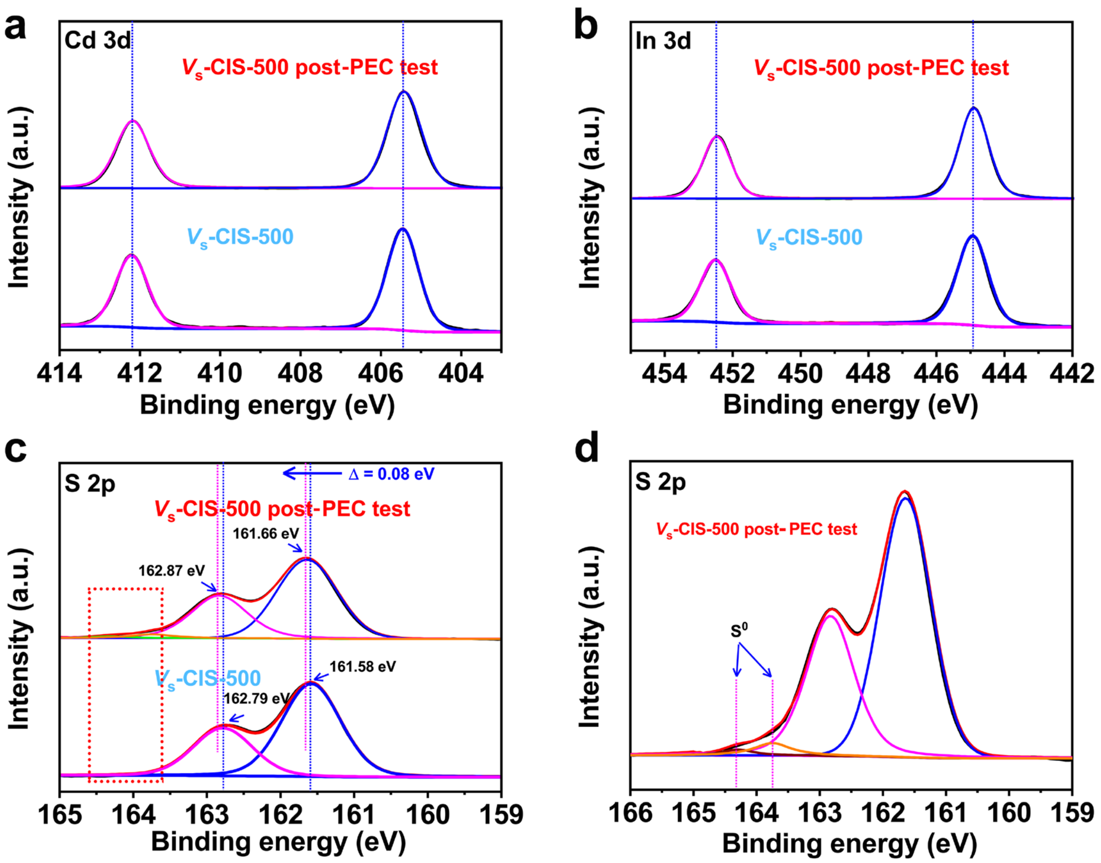

Supplementary Figure 11. **XPS spectra to illustrate the structural stability.** Core-level XPS spectra comparison of pristine V_s-CIS-500 photoanode and the photoanode post-PEC test for 15 h in 0.35 M Na_2SO_3 and 0.25 M Na_2S mixed solution. (**a**) Cd 3d, (**b**) In 3d, and (**c-d**) S 2p.

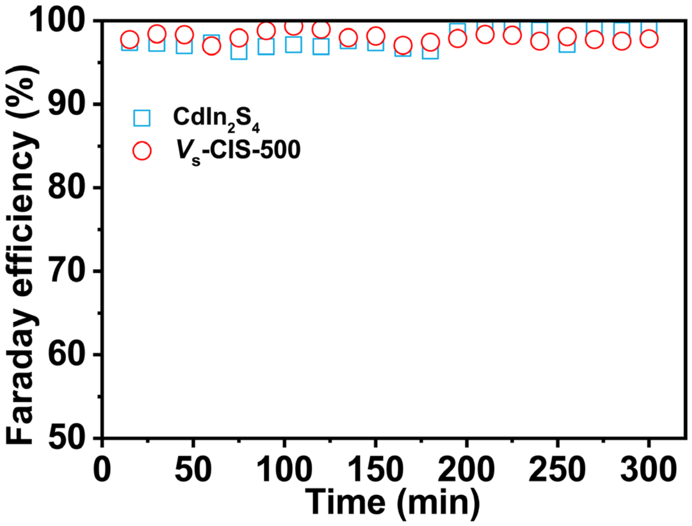

Supplementary Figure 12. Faradaic efficiency of V_s-CIS-500 photoanode measured at 0 V vs. Ag/AgCl electrode.

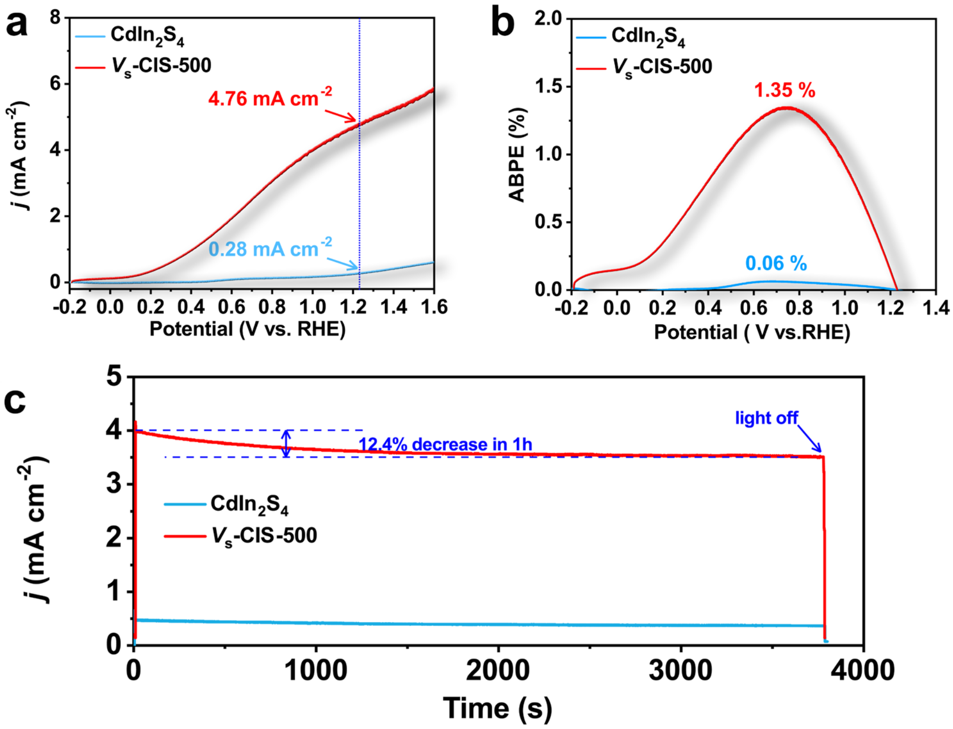

Supplementary Figure 13. **The PEC performances of the $CdIn_2S_4$ and V_s-CIS-500 photoanodes in Na_2SO_4 solution**. The (**a**) current-voltage *(J-V)* plots, (**b**) ABPE values, and (**c**) long-term stability of $CdIn_2S_4$ and V_s-CIS-500 photoanodes measured in 0.5 M Na_2SO_4 solution.

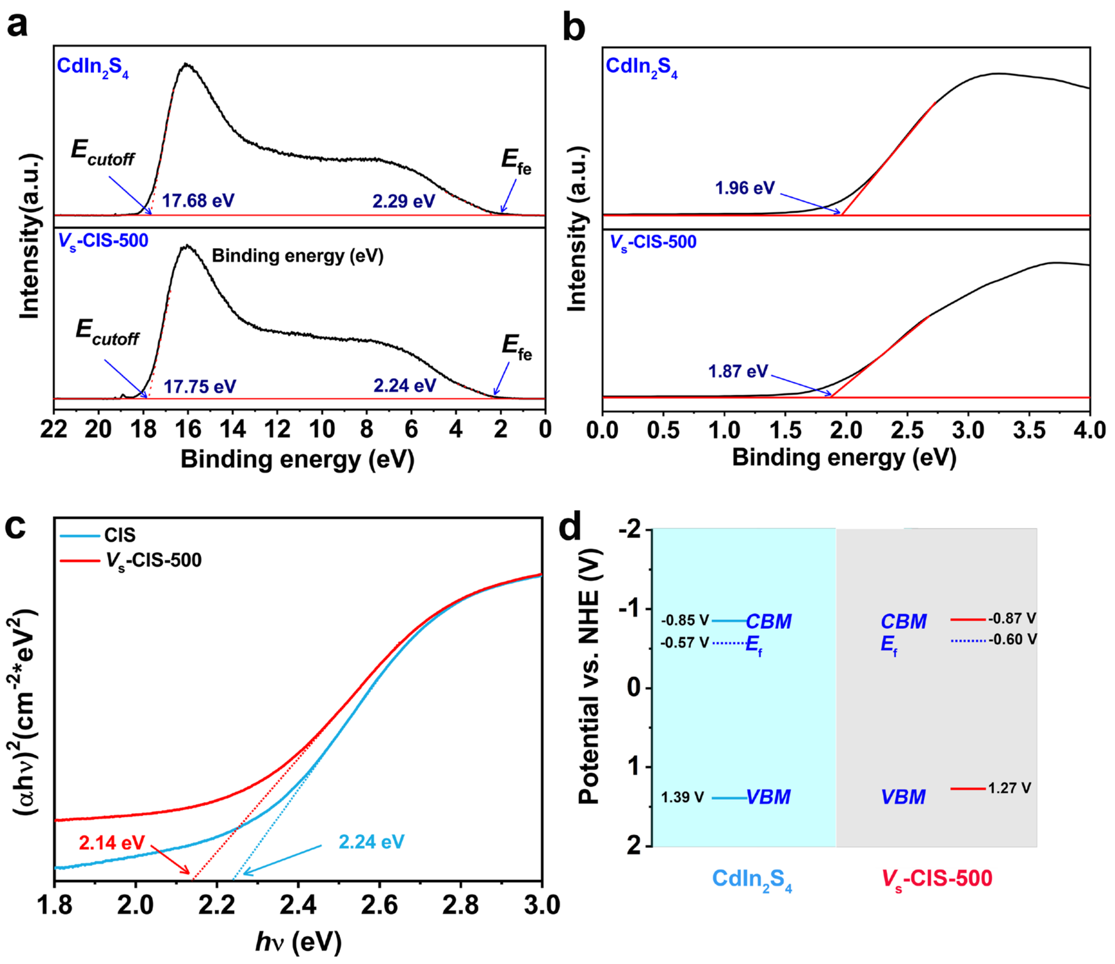

Supplementary Figure 14. Band structures of $CdIn_2S_4$ and V_s-CIS-500. The (**a**) UPS and (**b**) VB-XPS spectra, (**c**) the Tauc plots derived from UV-vis DRS spectra, and (**d**) the energy band diagram for $CdIn_2S_4$ and V_s-CIS-500. The E_{VB} is determined via the following equation: $E_{VB} = hv - (E_{cut} - E_{fe})$, where hv (21.22 eV) represents the incident photon energy from the He light source, E_{cut} represents the secondary electron cut-off edge, and E_{fe} represents the Fermi edge. After unit conversion relative to NHE (normal hydrogen electrode), that is, 0 V versus NHE equals to -4.44 eV, the conduction band edges (E_{CB}) are further calculated ($E_{CB} = E_{VB} - E_g$). Furthermore, the distances between the VB edge and Fermi levels (E_F) are determined by valence-band XPS spectra, and the E_F values are further confirmed. Detailed values of E_{VB}, E_{CB}, and E_F for $CdIn_2S_4$ and V_s-CIS-500 are supplemented in Supplementary Fig. 14d.

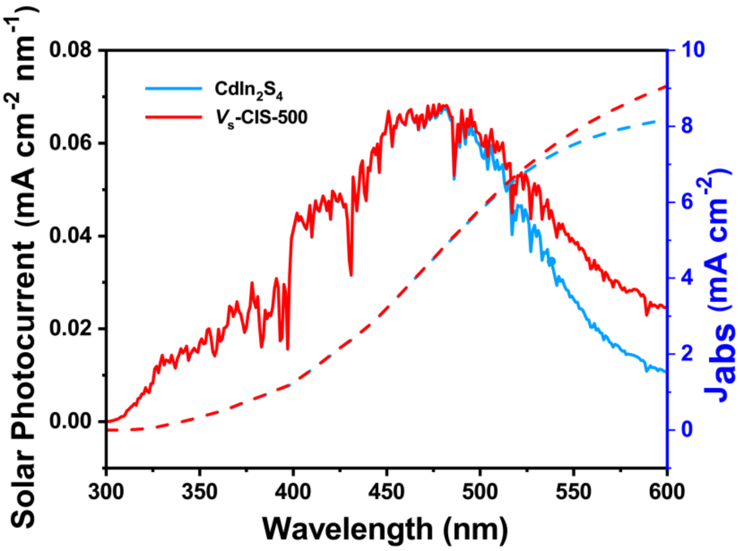

Supplementary Figure 15. Evaluation for the light-harvesting efficiency. The photon absorption rates (J_{abs}) of $CdIn_2S_4$ and V_s-CIS-500 photoanodes.

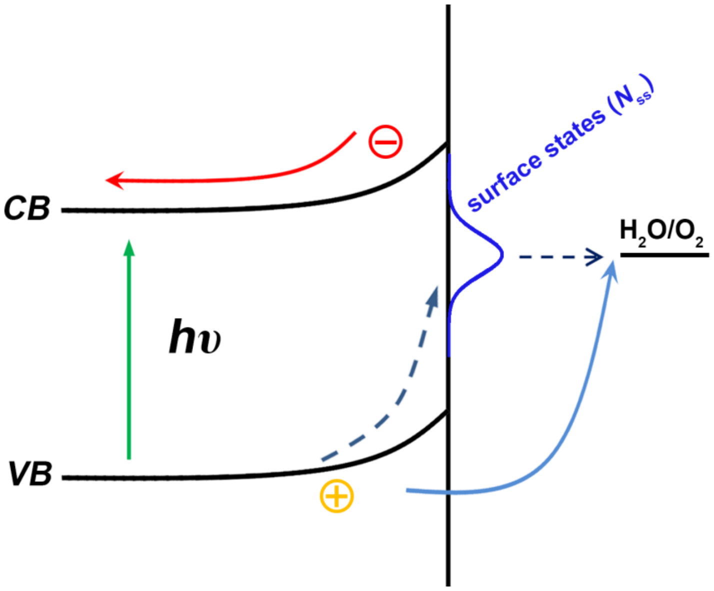

Supplementary Figure 16. **Surface models for hole transfer.** The simplified model of the elementary processes in a $CdIn_2S_4$ photoanode without cocatalyst.

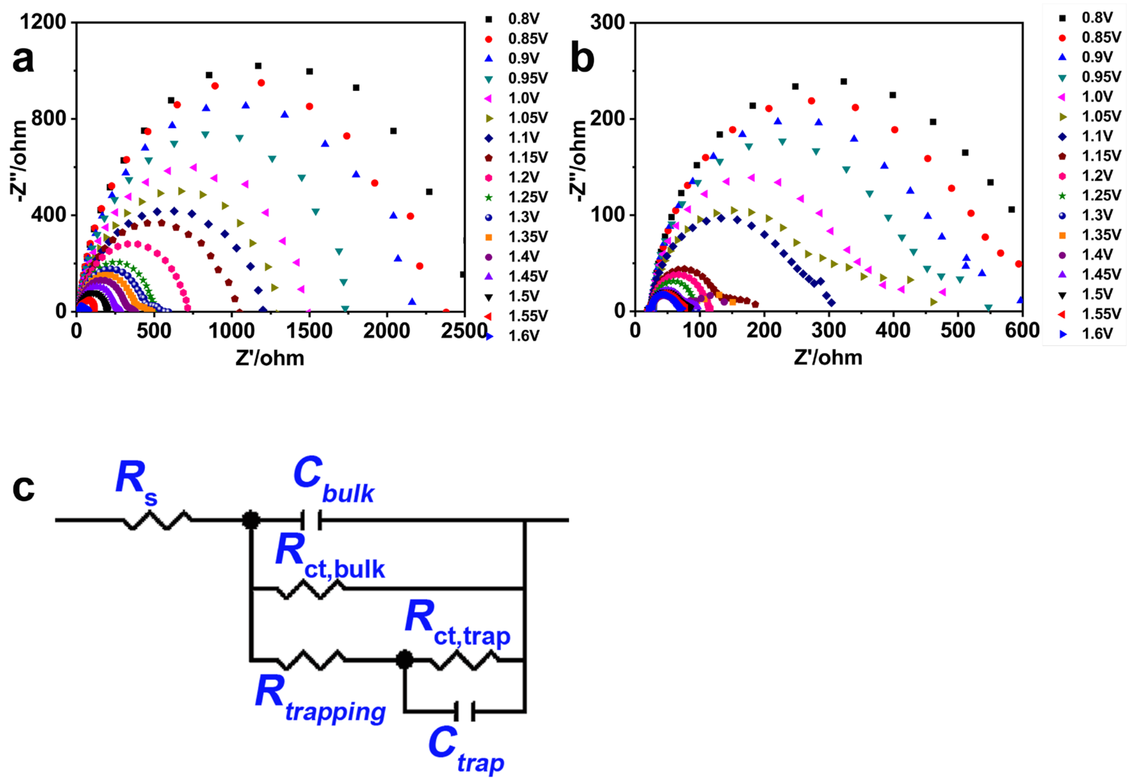

Supplementary Figure 17. **The EIS results**. The EIS plots of (**a**) $CdIn_2S_4$ and (**b**) V_s-CIS-500 photoanodes at different applied bias under constant light (430–720 nm, 100 mW cm^{-2}) illumination, as well as (**c**) the corresponding equivalent circuit.

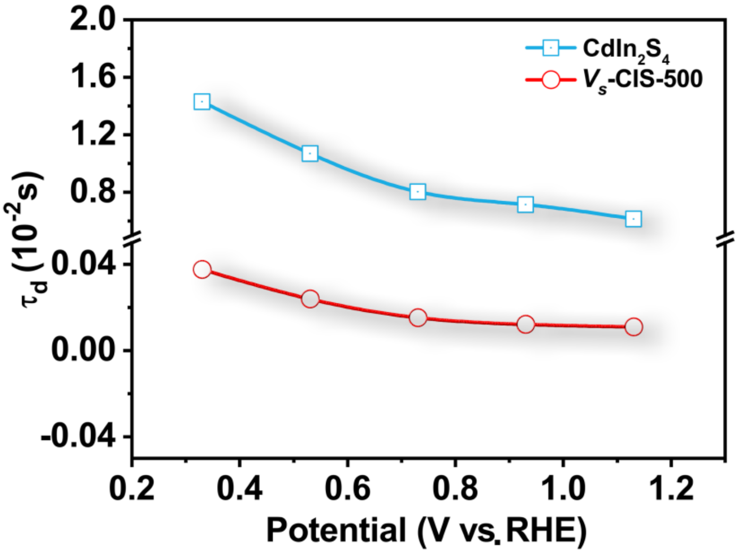

Supplementary Figure 18. Charge transfer and recombination kinetics. The plot of the average lifetime of photoinduced electrons (τ_d) vs. potential.

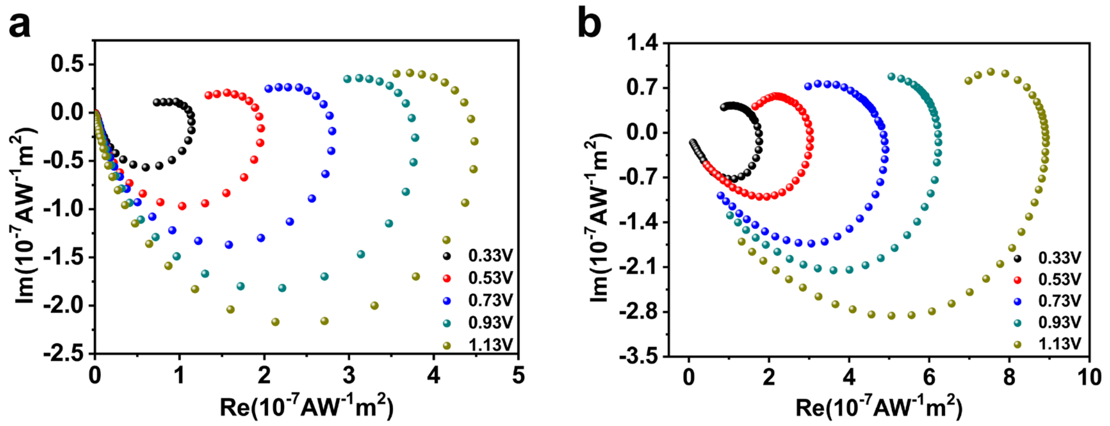

Supplementary Figure 19. **The IMPS results.** The IMPS spectra of (a) $CdIn_2S_4$ and (b) V_s-CIS-500 photoanodes at different applied bias.

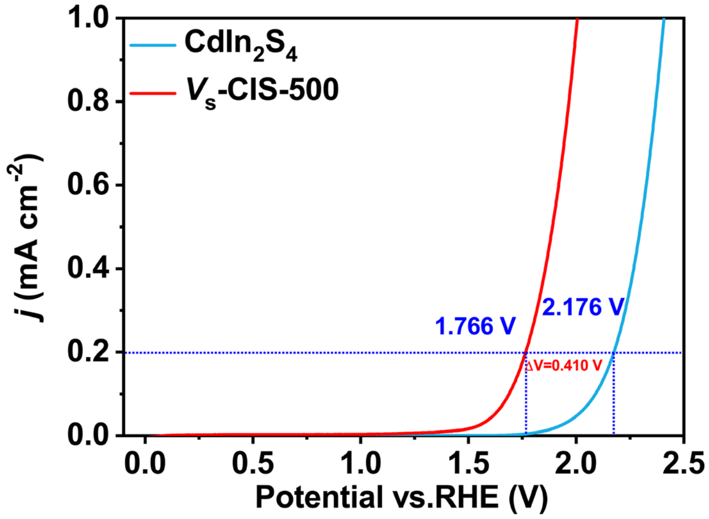

Supplementary Figure 20. Experimental overpotentials determination for the $CdIn_2S_4$ and V_s-CIS-500 photoanodes. The *J*-*V* plots of $CdIn_2S_4$ and V_s-CIS-500 photoanodes in the dark in 0.5 M Na_2SO_4 solution. The onset potentials of $CdIn_2S_4$ and V_s-CIS-500 photoanodes in the dark are determined at a photocurrent density of 0.2 mA cm^{-2}, which are 2.176 and 1.766 V, respectively, corresponding to η_{OER} of 0.946 and 0.536 V, consistent with the trend of theoretical values.

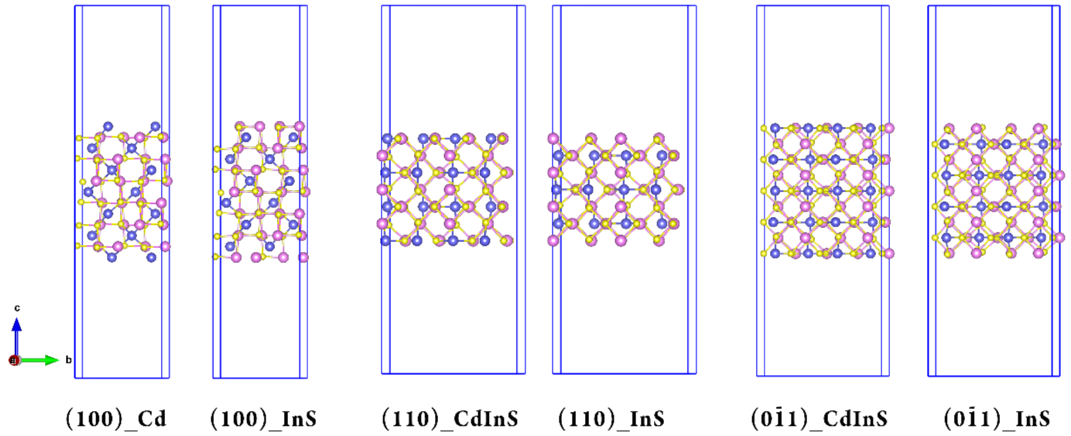

Supplementary Figure 21. Models for surface energy calculations. Slab models constructed for (100), (110), and (0$\bar{1}$1) crystal planes of $CdIn_2S_4$.

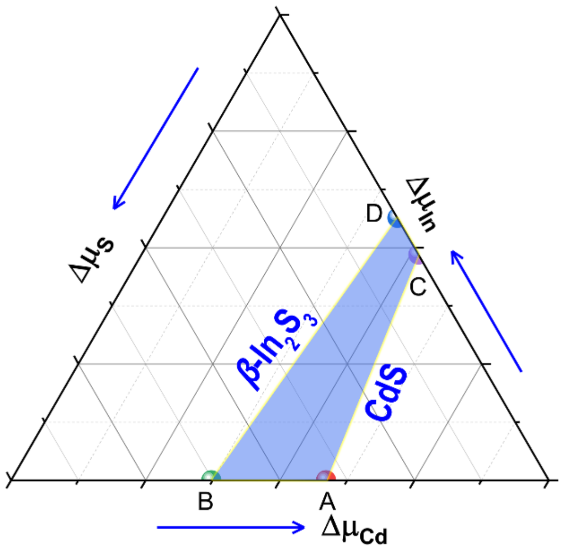

Supplementary Figure 22. **Possible chemical potentials for $CdIn_2S_4$.** Range of possible stoichiometric regimes (shaded area) for the chemical potentials of Cd, In, and S to sustain stable growth of $CdIn_2S_4$.

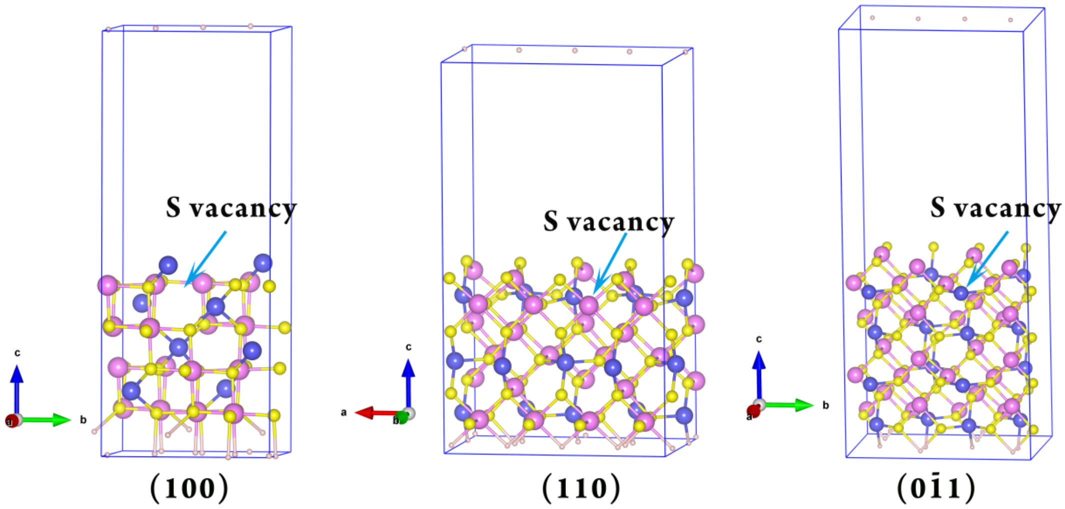

Supplementary Figure 23. Models for sulfur vacancies calculations. Slab models constructed for the surface sulfur vacancy.

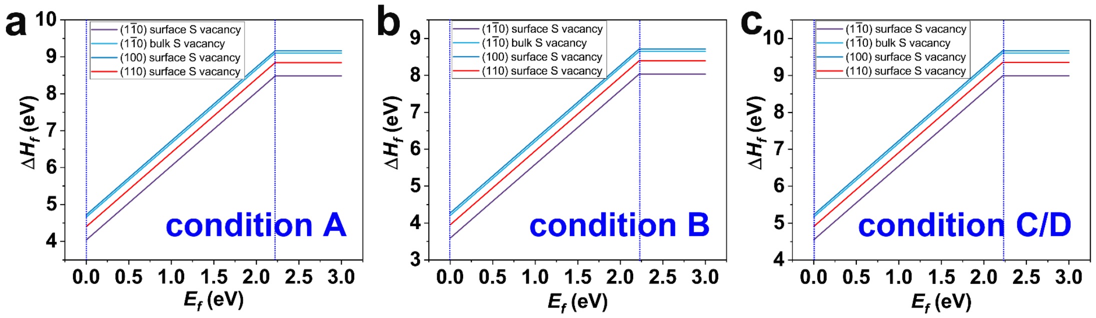

Supplementary Figure 24. Formation energies for sulfur vacancies in different chemical environments. Plots of formation energies of sulfur vacancy in various surfaces of $CdIn_2S_4$ vs. Fermi level in condition (**a**) A, (**b**) B, and (**c**) C/D.

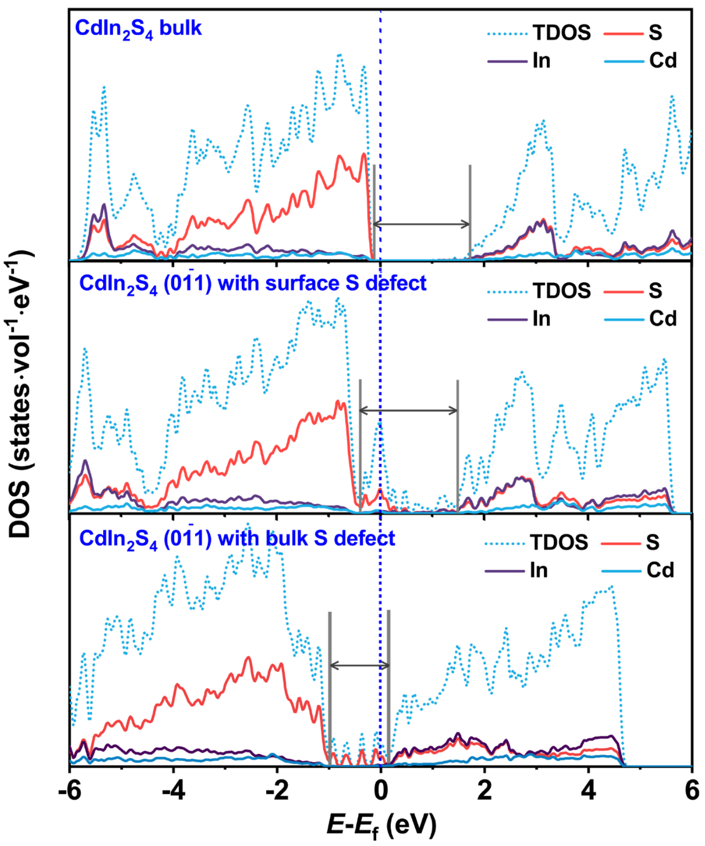

Supplementary Figure 25. **DOS plots.** The density of states for $CdIn_2S_4$ bulk, $CdIn_2S_4$ $(0\bar{1}1)$ with surface S and bulk vacancies.

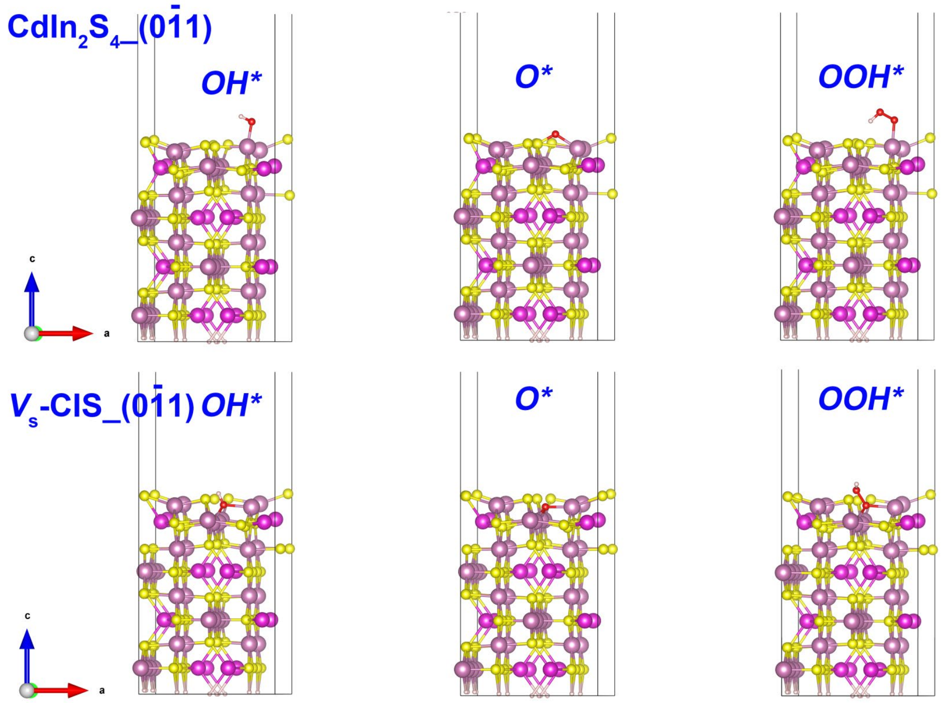

Supplementary Figure 26. Models for surface OER mechanism calculations. The optimized geometries of OH^*, O^*, and OOH^* intermediates absorbing on $CdIn_2S_4$-($0\bar{1}1$) and V_s-CIS-($0\bar{1}1$).

Supplementary Tables

Supplementary Table 1. The *J* and ABPE values of V_s-CIS-500 photoanode compared with other up-to-date metal sulfide-based photoanodes.

**Photoanodes**	**Photocurrent (mA cm^{-2} at 1.23V vs RHE)**	**ABPE (%)**	**Electrolyte**	**refs**
TiO_2/CdS/Co–Pi	1.1	0.48%	0.1 M sodium phosphate	1
Cu_7S_4/TiO_2/CoCr-LDH	2.04	0.58%	0.5 M Na_2SO_4	2
$TiO_2/CdS/MoS_2$	3.25	Not reported	0.25 M Na_2S and 0.35 M Na_2SO_3	3
CdS@$CdIn_2S_4$	5.5	0.45%	0.25 M Na_2S and 0.35 M Na_2SO_3	4
Zn/In:SnS_2	0.23	Not reported	0.5 M Na_2SO_4	5
CdS/SnS_x	4.5	Not reported	1 M phosphate buffer + 1 M Na_2SO_3 (pH 7)	6
	2.6		0.5 M Na_2SO_4	
CdS/SnS_x	1.59	Not reported	0.5 M Na_2SO_4	7
TiS_3 NR arrays	~14	Not reported	0.5 M potassium phosphate + 1 M Na_2SO_3	8
V_s-$CdIn_2S_4$	4.76	1.35%	0.5 M Na_2SO_4	This work
V_s-$CdIn_2S_4$	5.73	2.49%	0.25 M Na_2S and 0.35 M Na_2SO_3	This work

Supplementary Table 2. The *J* and ABPE values of $CdIn_2S_4$ and V_s-CIS-500 photoanodes compared with other up-to-date most promising photoanodes.

**Photoanodes**	**Single-photon absorber or composites**	**Photocurrent (mA cm^{-2} at 1.23V vs RHE)**	**ABPE (%)**	**refs**
$BiVO_4$, bandgap: 2.4eV<br>Theoretical STH: 9.2%	nanocone/Mo: $BiVO_4$<br>nanocone/Mo: $BiVO_4$/Fe(Ni)OOH	4.18<br>5.82	0.74<br>2.05	9
	$BiVO_4$<br>NiOOH/FeOOH/CQD/ $BiVO_4$	1.35<br>5.99	0.25%<br>2.29%	10
	$BiVO_4$<br>β-FeOOH/$BiVO_4$	1.2<br>4.3	0.1%<br>0.71%	11
Ta_3N_5, bandgap: 2.07eV<br>Theoretical STH: 15.9%	Ta_3N_5<br>complex 2/complex 1/$Ni(OH)_x$/Fh/TiO_x/Ta_3N_5	0.9<br>12.1	0.17%<br>2.5%	12
	Sc-Ta_3N_5<br>Sc-Ta_3N_5/$Co(OH)_x$	1.2<br>4.9	-<br>0.50%	13
	Ta_3N_5: Mg+Zr<br>CoO_x-FeO_x/ Ta_3N_5:Mg+Zr	0.45<br>2.3	-<br>0.59%	14
Fe_2O_3, bandgap: 2.1eV<br>Theoretical STH: 15.3%	Fe_2O_3	0.2	Not reported	15
	Ti: Fe_2O_3<br>Co-Pi/Co_3O_4/Ti:Fe_2O_3	1.2<br>2.7	0.17%<br>0.43%	16
	Fe_2O_3 NT<br>Fe_2O_3 NT-FeOOH/NiOOH	1.2<br>2.0	0.1%<br>0.29%	17
	Fe_2O_3-PN<br>Fe_2O_3-PN/CoPi	1.06<br>1.6	0.1%<br>0.21%	18

$CdIn_2S_4$, bandgap: 2.24eV<br>Theoretical STH: 12.39%	$CdIn_2S_4$ (Na_2SO4)	0.28	0.06%	This work
V_s-$CdIn_2S_4$, bandgap: 2.14eV<br>Theoretical STH: 15.24%	V_s-$CdIn_2S_4$(Na_2SO_4)	4.76	1.35%	This work

Supplementary Table 3. Structure parameters for surface energy calculations.

**Surfaces**	**Numbers of atoms**	**Surface areas/Å^2**	**K-mesh**
**(100)_Cd**	$Cd_{14}In_{24}S_{48}$	117.7	5×5×1
**(100)_InS**	$Cd_{12}In_{28}S_{48}$	117.7	5×5×1
**(110)_CdInS**	$Cd_{16}In_{28}S_{56}$	166.4	5×3×1
**(110)_InS**	$Cd_{12}In_{28}S_{48}$	166.4	5×3×1
**($0\bar{1}1$) _CdInS**	$Cd_{16}In_{28}S_{56}$	166.4	5×3×1
**($0\bar{1}1$) _InS**	$Cd_{12}In_{28}S_{56}$	166.4	5×3×1

Supplementary Table 4. Surface energies of $(0\bar{1}1)$, (100), and (110) crystal planes calculated in S rich condition.

**Surfaces**	**γ (mJ m^{-2}) in condition C**	**γ (mJ m^{-2}) in condition D**	**Average γ**
**(100)_Cd**	826.9	867.6	847.3
**(100)_InS**	1288.9	1248.4	1268.7
**(110)_CdInS**	584.1	612.9	598.5
**(110)_InS**	893.8	865.1	879.5
**$(0\bar{1}1)$ _CdInS**	584.1	612.9	598.5
**$(0\bar{1}1)$ _InS**	599.7	570.9	585.3

Supplementary Table 5. Total energies of clean $CdIn_2S_4$-$(0\bar{1}1)$ and *V_s*-CIS-$(0\bar{1}1)$ as well as the energies of the most stable absorption geometries for O^*, OH^*, and OOH^* intermediates.

**Surfaces**	***E*(*)/eV**	***E*(O*)/eV**	***E*(OH*)/eV**	***E*(OOH*)/eV**
**Clean $CdIn_2S_4$-$(0\bar{1}1)$**	-466.866	-473.331	-477.996	-482.487
**V_s-CIS-$(0\bar{1}1)$**	-459.053	-465.764	-471.066	-475.296

Supplementary Table 6. Frequencies of adsorbed species on $CdIn_2S_4$-($0\bar{1}1$) and V_s-CIS-($0\bar{1}1$).

**Adsorbed species on $CdIn_2S_4$-($0\bar{1}1$)**	**Frequency (cm^{-1})**
**O***	695.9, 406.9, 212.1
**OH***	3806.4, 792.2, 514.9,137.8,123.8,79.7
**OOH***	3051.9, 1422.6, 949.3, 586.4, 464.9, 218.7, 135.9, 107.9
**Adsorbed species on V_s-CIS-($0\bar{1}1$)**	**Frequency (cm^{-1})**
**O***	461.8, 394.8, 326.1
**OH***	3720.5, 735.2, 519.2, 337.3, 248.9, 190.7
**OOH***	3747.6, 1266.1, 880.8, 349.3, 258.5, 191.2, 169.4, 122.3

Supplementary Table 7. Ground state energy calculated by DFT, ZPE and *TS* correction for H_2O molecular adsorbing on $CdIn_2S_4$-$(0\bar{1}1)$and V_s-CIS-$(0\bar{1}1)$.

	E_{DFT} (eV)	ZPE (eV)	TS (eV)	△G (eV)
**$CdIn_2S_4$_$(0\bar{1}1)$**	-466.866	0	0	
**V_s-$CdIn_2S_4$_$(0\bar{1}1)$**	-459.053	0	0	
**H_2O**	-14.223	0.56	0.67	
**$CdIn_2S_4$_$(0\bar{1}1)$_H_2O**	-483.003	0.729	0.175	-1.251
**V_s-$CdIn_2S_4$_$(0\bar{1}1)$_H_2O**	-474.417	0.679	0.073	-0.425

Supplementary Notes

Supplementary Note 1. Surface energy calculations for $CdIn_2S_4$.

Given the effect of truncated atoms on the surface energy, (100), (110), and $(0\bar{1}1)$ crystal planes with variable truncated atoms were taken into account. As shown in Supplementary Figure 21 and Supplementary Table 3, the slab models were constructed to calculate their surface energies.

The chemical potential $\mu_{CdIn_2S_4}^{slab}$ of a condensed and stoichiometric phase of $CdIn_2S_4$ is written as a sum of the chemical potential of each species within the crystal:

$$\mu_{CdIn_2S_4}^{slab} = \mu_{Cd} + 2\mu_{In} + 4\mu_{S} \tag{1}$$

At 0K and constant pressure, the chemical potential of the surface in equilibrium with the chemical potential of bulk gives $\mu_{CdIn_2S_4}^{slab} = E_{BiOCl}^{bulk}$. Thus, according to our constructed slab models, the surface energy of (100), (110), and $(0\bar{1}1)$ crystal planes of $CdIn_2S_4$ can be expressed as:

$$\gamma_{100\_Cd} = \frac{1}{2A}(E_{slab}^{relax} - 12\mu_{CdIn_2S_4} - 2\mu_{Cd}) \tag{2}$$

$$\gamma_{100\_InS} = \frac{1}{2A}(E_{slab}^{relax} - 12\mu_{CdIn_2S_4} - 4\mu_{In}) \tag{3}$$

$$\gamma_{110\_CdInS} = \frac{1}{2A}(E_{slab}^{relax} - 14\mu_{CdIn_2S_4} - 2\mu_{Cd}) \tag{4}$$

$$\gamma_{110\_InS} = \frac{1}{2A}(E_{slab}^{relax} - 12\mu_{CdIn_2S_4} - 4\mu_{In}) \tag{5}$$

$$\gamma_{0\bar{1}1\_CdInS} = \frac{1}{2A}(E_{slab}^{relax} - 14\mu_{CdIn_2S_4} - 2\mu_{Cd}) \tag{6}$$

$$\gamma_{0\bar{1}1\_InS} = \frac{1}{2A}(E_{slab}^{relax} - 14\mu_{CdIn_2S_4} + 2\mu_{Cd}) \tag{7}$$

The surface energy is highly relevant to the chemical potentials of μ_{Cd}, μ_{In}, and μ_{S}. Therefore, the upper and lower boundaries for the elemental chemical potentials involved in $CdIn_2S_4$ are investigated. The chemical potentials represent the growth conditions of atoms, which must be carefully treated to investigate the relative stability of introduced defects. Under thermal equilibrium growth condition, $CdIn_2S_4$ should satisfy Supplementary eq 8. Moreover, precipitation of secondary phases such as CdS and β-In_2S_3 (β-In_2S_3 is the most stable phase among α-, β-, and γ-In_2S_3 under room

temperature, with the smallest formation enthalpy[19]) should be excluded. Simultaneously, the chemical potential of each element must not be larger than the corresponding chemical potential of the bulk element. In equations, all conditions could be summarized as follows.

$$\Delta\mu_{\mathrm{Cd}} + 2\Delta\mu_{\mathrm{In}} + 4\Delta\mu_{\mathrm{S}} = H^{\mathrm{f}}_{\mathrm{CdIn_2S_4}} \quad (8)$$

$$\Delta\mu_{\mathrm{Cd}} + \Delta\mu_{\mathrm{S}} \leq H^{\mathrm{f}}_{\mathrm{CdS}} \quad (9)$$

$$2\Delta\mu_{\mathrm{In}} + 3\Delta\mu_{\mathrm{S}} \leq H^{\mathrm{f}}_{\beta\text{-}\mathrm{In_2S_3}} \quad (10)$$

$$\Delta\mu_{\mathrm{Cd}} \leq 0\,;\Delta\mu_{\mathrm{In}} \leq 0\,;\Delta\mu_{\mathrm{S}} \leq 0 \quad (11)$$

According to the equations described above, the atomic chemical potentials for Cd, In, and S atoms to possibly generate $CdIn_2S_4$ are indicated by the shaded area, as shown in Supplementary Figure 22. The upper boundary is given by the equations $\Delta\mu_{\mathrm{Cd}} = H^{\mathrm{f}}_{\mathrm{CdS}} - \Delta\mu_{\mathrm{S}}$ and $\Delta\mu_{\mathrm{In}} = (H^{\mathrm{f}}_{\mathrm{CdIn_2S_4}} - H^{\mathrm{f}}_{\mathrm{CdS}} - 3\Delta\mu_{\mathrm{S}})/2$. Therefore, point A corresponds to the condition in equilibrium with In bulk ($\Delta\mu_{\mathrm{In}}$ = 0 eV) and poor S possible ($\Delta\mu_{\mathrm{S}}$ = –0.510 eV, $\Delta\mu_{\mathrm{Cd}}$ = –0.721 eV). Point C is S rich ($\Delta\mu_{\mathrm{S}}$ = 0 eV) but poorest in Cd, with $\Delta\mu_{\mathrm{Cd}}$ = –1.231 eV and $\Delta\mu_{\mathrm{In}}$ = –1.589eV. The lower boundary is given by the equations $\Delta\mu_{\mathrm{In}} = (H^{\mathrm{f}}_{\mathrm{CdIn_2S_4}} - H^{\mathrm{f}}_{\mathrm{CdS}} - 3\Delta\mu_{\mathrm{S}})/2$ and $\Delta\mu_{\mathrm{Cd}} = H^{\mathrm{f}}_{\mathrm{CdIn_2S_4}} - H^{\mathrm{f}}_{\beta\text{-}\mathrm{In_2S_3}} - \Delta\mu_{\mathrm{S}}$. Point B corresponds to equilibrium with In bulk and poorest S possible, where $\Delta\mu_{\mathrm{S}}$ = –0.960 eV and $\Delta\mu_{\mathrm{Cd}}$ = –0.570 eV. Finally, point D corresponds to the condition poorest in In with $\Delta\mu_{\mathrm{s}}$ = 0, $\Delta\mu_{\mathrm{In}}$= –1.440 eV and $\Delta\mu_{\mathrm{Cd}}$ = –1.530 eV.

Given the excessive thioacetamide in the experimental condition which indicates the S rich in chemical potential, the surface energies for (100), (110), and $(0\bar{1}1)$ crystal planes of $CdIn_2S_4$ are calculated in condition C and D, and the final surface energies are their average value in the two conditions (Supplementary Table 4), illustrating that the exposed surface with In and S as truncated atoms are more stable in $(0\bar{1}1)$, which is in good agreement with the HAADF-STEM image and provides the atomic structure of $(0\bar{1}1)$ for simulating the surface OER process.

Supplementary Note 2. Formation energy calculations of sulfur vacancies in $CdIn_2S_4$.

Based on the above elemental chemical potential range, the formation energy of sulfur vacancies (V_S^q = 0, 1+ and 2+) is investigated. According to eq 15, sulfur vacancies with 2+ charge state should have the lowest formation energy, which indicates $CdIn_2S_4$ with 2+ charge state sulfur vacancy is the most stable. Therefore, we only consider the formation energy of 2+ charge state sulfur vacancy in the surface or bulk of $CdIn_2S_4$. As shown in Supplementary Figure 23, surface sulfur vacancies in the $(0\bar{1}1)$ crystal plane have the lowest formation energy in all possible stoichiometric regimes for the atom chemical potentials of Cd, In, and S. Therefore, we consider the sulfur vacancy on the surface of the $(0\bar{1}1)$ crystal plane is responsible for the change of photoelectrochemical property of the $CdIn_2S_4$ photoanode.

Besides, according to the DOS plots (Supplementary Fig. 25), bulk sulfur vacancies bring about big bandgap reduction for $CdIn_2S_4$. Whereas, the bandgap varies little after introducing sulfur vacancies as illustrated by the UV-vis DRS spectra (Fig. 3A, Supplementary Fig. 14c), which also illustrates that the defective sites should be surface sulfur vacancies other than bulk vacancies.

Supplementary Note 3. OER mechanism on the $(0\bar{1}1)$ plane of $CdIn_2S_4$ with and without sulfur vacancies.

The OER process in the four electrons pathways is summarized using the following elementary steps[20].

$$* + H_2O(l) \rightarrow OH^* + (H^+ + e^-) \tag{12}$$

$$OH^* \rightarrow O^* + (H^+ + e^-) \tag{13}$$

$$O^* + H_2O \rightarrow OOH^* + (H^+ + e^-) \tag{14}$$

$$OOH^* \rightarrow * + O_2 + (H^+ + e^-) \tag{15}$$

Where the asterisk (*) stands for the active site on the catalyst, while O*, OH*, and OOH* represent the intermediates during the OER evolution.

For each step, the reaction Gibbs free energy (ΔG) is defined as the difference between free energies of the initial and final states and is given by the expression[20],

$$\Delta G = \Delta E + \Delta ZPE - T\Delta S - eU \tag{16}$$

Where the ΔE is the reaction energy of reaction and product molecules absorbed on catalyst surface obtained from DFT calculations, ΔZPE is the difference of zero-point energy, T equals to 298.15 K, ΔS is the entropy change, and U is the potential applied at the electrode. Of note, total energies of clean CIS-$(0\bar{1}1)$ and V_s-CIS-$(0\bar{1}1)$, as well as the energies of the most stable O^*, OH^*, OOH^* species absorption geometries, are provided in **Supplementary Table 3**. The energies of H_2O(l) and H_2 (g) are referenced to Nørskov's work[20], and frequencies of adsorbed species, which is calculated for the zero-point energy (ZPE) corrections, are supplemented in **Supplementary Table 4**.

The overpotential (η_{OER}) for OER can be obtained by Supplementary eq 17[20].

$$\eta_{OER} = \frac{\max\{\Delta G_1, \Delta G_2, \Delta G_3, \Delta G_4\}}{e} - 1.23V \tag{17}$$

Where ΔG_1, ΔG_2, ΔG_3, and ΔG_4 are the free energies of reactions (Supplementary eq

12-15), respectively.

The adsorption energies of H_2O molecules on $CdIn_2S_4$-$(0\bar{1}1)$ and V_s-CIS-$(0\bar{1}1)$ are calculated according to the following equation, and detailed data are supplemented in Supplementary Table 5.

$$\Delta G = G_{\mathrm{substrate+H_2O}} - G_{\mathrm{substrate}} - G_{\mathrm{H_2O}} \tag{18}$$

Where $G_{\mathrm{substrate+H2O}}$ is the energy of optimized absorbing geometry of H_2O molecules on $CdIn_2S_4$-$(0\bar{1}1)$ or V_s-CIS-$(0\bar{1}1)$ surface, $G_{\mathrm{substrate}}$ is the energy of $CdIn_2S_4$-$(0\bar{1}1)$ or V_s-CIS-$(0\bar{1}1)$ surface and G_{H2O} is the energy of H_2O molecules at 0.024 bar and 300 K.

Supplementary References

1. Ai G, Li H, Liu S, Mo R, Zhong J. Solar water splitting by TiO_2/CdS/Co–Pi nanowire array photoanode enhanced with Co–Pi as hole transfer relay and CdS as light absorber. *Adv. Funct. Mater.* **25**, 5706-5713 (2015).

2. Ran L, Yin L. Ternary Hierarchical Cu_7S_4/TiO_2/CoCr-LDH Heterostructured Nanorod Arrays with Multiphase Reaction Interfaces for More Efficient Photoelectrochemical Water Splitting. *Adv. Mater. Interfaces* **6**, 1800970 (2019).

3. Bhat SS*, et al.* Substantially enhanced photoelectrochemical performance of TiO_2 nanorods/CdS nanocrystals heterojunction photoanode decorated with MoS_2 nanosheets. *Appl. Catal. B: Environ.* **259**, 118102 (2019).

4. Song J-P, Yin P-F, Mao J, Qiao S-Z, Du X-W. Catalytically active and chemically inert $CdIn_2S_4$ coating on a CdS photoanode for efficient and stable water splitting. *Nanoscale* **9**, 6296-6301 (2017).

5. Meng L, Wang S, Cao F, Tian W, Long R, Li L. Doping-induced amorphization, vacancy, and gradient energy band in SnS_2 nanosheet arrays for improved photoelectrochemical water splitting. *Angew. Chem. Inter. Ed.* **58**, 6761-6765 (2019).

6. Giri B*, et al.* Balancing light absorption and charge transport in vertical SnS_2 nanoflake photoanodes with stepped layers and large intrinsic mobility. *Advanced Energy Materials* **9**, 1901236 (2019).

7. Fu Y*, et al.* Phase-modulated band alignment in CdS nanorod/SnS_x nanosheet hierarchical heterojunctions toward efficient water splitting. *Adv. Funct. Mater.* **28**, 1706785 (2018).

8. Tian Z*, et al.* Enhanced charge carrier lifetime of TiS_3 photoanode by introduction of S_2^{2-} vacancies for efficient photoelectrochemical hydrogen evolution. *Adv. Funct. Mater.*, 2001286 (2020).

9. Qiu Y*, et al.* Efficient solar-driven water splitting by nanocone $BiVO_4$-perovskite tandem cells. *Sci. Adv.* **2**, e1501764 (2016).

10. Ye K-H*, et al.* Carbon quantum dots as a visible light sensitizer to significantly increase the solar water splitting performance of bismuth vanadate photoanodes. *Energy Environ. Sci.* **10**, 772-779 (2017).

11. Zhang B, Wang L, Zhang Y, Ding Y, Bi Y. Ultrathin FeOOH nanolayers with abundant oxygen vacancies on $BiVO_4$ photoanodes for efficient water oxidation. *Angew. Chem. Int. Ed.* **57**, 2248-2252 (2018).

12. Liu G, *et al.* Enabling an integrated tantalum nitride photoanode to approach the theoretical photocurrent limit for solar water splitting. *Energy Environ. Sci.* **9**, 1327-1334 (2016).

13. Pei L, *et al.* Oriented Growth of Sc-Doped Ta_3N_5 Nanorod Photoanode Achieving Low-Onset-Potential for Photoelectrochemical Water Oxidation. *ACS Appl. Energy Mater.* **1**, 4150-4157 (2018).

14. Seo J, *et al.* Mg-Zr cosubstituted Ta_3N_5 photoanode for lower-onset-potential solar-driven photoelectrochemical water splitting. *J. Am. Chem. Soc.* **137**, 12780-12783 (2015).

15. Wang G, *et al.* Enhancing and stabilizing α-Fe_2O_3 photoanode towards neutral water oxidation: Introducing a dual-functional NiCoAl layered double hydroxide overlayer. *J. Catal.* **359**, 287-295 (2018).

16. Yi SS, Wulan BR, Yan JM, Jiang Q. Highly efficient photoelectrochemical water splitting: surface modification of cobalt-phosphate-loaded Co_3O_4/Fe_2O_3 p–n heterojunction nanorod arrays. *Adv. Funct. Mater.* **29**, 1801902 (2019).

17. Feng C, Fu S, Wang W, Zhang Y, Bi Y. High-Crystalline and High-Aspect-Ratio Hematite Nanotube Photoanode for Efficient Solar Water Splitting. *Appl. Catal. B-Environ.* 117900 (2019).

18. Liu G, *et al.* Porous versus compact hematite nanorod photoanode for high performance photoelectrochemical water oxidation. *ACS Sustainable Chem. Eng.* **7**, **13**, 11377-11385, (2019).

19. Gao Z, Liu J, Wang H. Investigation on growth of In_2S_3 thin films by chemical bath deposition. *Mat. Sci. Semicon. Proc.* **15**, 187-193 (2012).

20. Man IC, *et al.* Universality in Oxygen Evolution Electrocatalysis on Oxide Surfaces. *ChemCatChem* **3**, 1159-1165 (2011).
