# Supplementary material for: Highly active deficient ternary sulfide photoanode for photoelectrochemical water splitting
Source: Nat Commun. 2020 Jun 17;11:3078. doi: 10.1038/s41467-020-16800-w (PMC7299993; doi:10.1038/s41467-020-16800-w)
Supplement: Supplementary file 2 — Peer Review File [file 41467_2020_16800_MOESM2_ESM.docx]

Peer Review Information

**Journal:** Nature Communications

**Manuscript title:** Click here to enter text.
Highly active deficient ternary sulfide photoanode for photoelectrochemical water splitting

**Corresponding author name(s):** Dr Yuguo Xia

**Editorial notes:**

**Reviewer comments & decisions:**

**Reviewer comments, first version:**

Reviewers' comments:

Reviewer #1 (Remarks to the Author):

This manuscript investigates CdInS4, a ternary metal sulfide, for its application as photoanode and its photoelectrochemical performance impact of introducing sulfur vacancies. They conduct experimental characterization supported with theoretical calculations.

A detailed study experimental study shows that ternary sulfide is a potential candidate as a photoanode for photoelectrochemical water splitting. The surface vacancies, due to missing S, results in improved photocurrent.

The paper has a good quality of research, with substantial experimental details. To conclude, the content of the paper is publishable with edits.

There are a few suggestions to improve the paper:
1. Theoretical investigation is missing basic information, like how the calculations were set up. Please consider including simulation details.
2. The way details of the experiment set up are described in the manuscript, they should include technical details regarding simulations.
3. From TDOS calculations of Fig. S21 it seems that S vacancies induce additional trap states in the bandgap. It might be useful to see references seeing such effect (either experimentally or theoretically). Also, explain how that leads to an improved photocurrent


Reviewer #2 (Remarks to the Author):

In this manuscript, Wang et.al prepared and investigated the novel deficient ternary metal sulfide (CdIn2S4) as photoanodes for water oxidation. Through mild annealing treatment (Ar/H2, 500 oC), the surface S vacancies were introduced to the CdIn2S4 photoanode. Higher photocurrent was observed on the photoanode relative to the pristine CdIn2S4 thin film. They attributed the enhanced activity of photoanode to the effect of surface S vacancies on the interfacial charge separation and transfer kinetics. They also demonstrated the restrained surface states distribution and the transformation of active sites after introducing surface S vacancies. However, many works related with this topic have been reported and the innovation is not new enough. This work can not be recommended for publication because of the following problems.
1. In the Introduction section, the selection reason of S as vacancies for improving the water oxidation activity of CdIn2S4 photoanode is not solidly introduced. Several typical works related to the S vacancies introduced photoanodes have been reported in recent years.
2. To introduce the S vacancies into photoanode, the CdIn2S4 was annealed with Ar/H2 flow at 500 oC. The Ar/H2 possesses reducibility, will the photoanode be reduced? The XPS date about S and O should be provided.
3. The author claimed when the photoanode was annealed at 500 oC, the In2O3 appeared, but the annealing flow is Ar/H2, why did oxides appear?
4. The dark photocurrent measured in 0.35 M Na2SO3 and 0.25 M Na2S mixed solution (pH =12.5) should be provided.
5. After the long-term J-t test, the authors should provide the XPS data to indicate its robust photoelectrochemical stability.
6. The instability of photoanode is due to the photocorrosion caused by holes collected in the photoanode surface, thus the long-term J-t test must be measured in Na2SO4.
7. The ηseperation and ηinjection was measured in the electrolyte solution with and without Na2SO3 as a hole scavenger in the Figure 2a, why the J-V plots was measured in 0.35 M Na2SO3 and 0.25 M Na2S mixed solution (pH =12.5).


Reviewer #3 (Remarks to the Author):

Reviewer's remarks on the manuscript “Highly active deficient ternary sulfide photoanode for photoelectrochemical water splitting”

The manuscript reports on photoelectrocatalytic activity of spinel CdIn2S4 photoanodes with artificially introduced sulfur vacancies in wster splitting. The authors report unprecedently high light-to-current and light-to-hydrogen conversion efficiencies along with absolutely unexpected stability of such S-deficient material both in the splitting of pure water where typically strong photocorrosion of sulfides occur and in solutions containing sulfide ions that can eliminate the surface vacancies. The authors provided very deep experimental and calculational background explaining this unusual stability of the S-deficient CdIn2S4 photoanodes due to the presence of undercoordinated Cd and IN atoms (that is S vacancies).
The spinel CdIn2S4 is only occasionally studied as a photocatalyst and even rarely - as a photoelectrocatalyst and therefore the material as well as the approach for generation of the sulfur vacancies can be considered as novel. The authors then performed extensively deep characterization of the photoactive phase including probably all imaginable (among appropriate ones) physical and physio-chemical methods that can be applied to extract information on such materials, including EPR verification of vacancies and their direct observation through HAADF STEM. Also, the electrochemical properties of the photoanode-electrolyte system are studied deeply and comprehensively. The authors also addressed the issues of the kinetics and mechanism of the interfacial hole transfer on the surface of S-deficient CdIn2S4 and provided a deep insight into this process which undoubtedly contributes to our knowledge of the photoanodic processes on sulfide semiconductors and will inspire deeper studies of both this particular semiconductor and other possible ternary spinel compounds as well as future searches for the most favorable co-catalysts of water reduction and oxidation with such materials. The paper is logically built and written with acceptable language, the figures are excellent and rationally distributed between the main text and SI. No particular critical comments appeared during the analysis of the manuscript. Therefore, it can be recommended for the publication in the present form.

**Author rebuttal, first version:**

Reviewers' comments:

Reviewer #1 (Remarks to the Author):

This manuscript investigates CdIn_2_S_4_, a ternary metal sulfide, for its application as photoanode and its photoelectrochemical performance impact of introducing sulfur vacancies. They conduct experimental characterization supported with theoretical calculations. A detailed study experimental study shows that ternary sulfide is a potential candidate as a photoanode for photoelectrochemical water splitting. The surface vacancies, due to missing S, results in improved photocurrent.
The paper has a good quality of research, with substantial experimental details. To conclude, the content of the paper is publishable with edits.
There are a few suggestions to improve the paper:
1. Theoretical investigation is missing basic information, like how the calculations were set up. Please consider including simulation details.
2. The way details of the experiment set up are described in the manuscript, they should include technical details regarding simulations.
3. From TDOS calculations of Fig. S21 it seems that S vacancies induce additional trap states in the bandgap. It might be useful to see references seeing such effect (either experimentally or theoretically). Also, explain how that leads to an improved photocurrent.

Reviewer #2 (Remarks to the Author):

In this manuscript, Wang et.al prepared and investigated the novel deficient ternary metal sulfide (CdIn_2_S_4_) as photoanodes for water oxidation. Through mild annealing treatment (Ar/H_2_, 500^o^C), the surface S vacancies were introduced to the CdIn_2_S_4_ photoanode. Higher photocurrent was observed on the photoanode relative to the pristine CdIn_2_S_4_ thin film. They attributed the enhanced activity of photoanode to the effect of surface S vacancies on the interfacial charge separation and transfer kinetics. They also demonstrated the restrained surface states distribution and the transformation of active sites after introducing surface S vacancies. However, many works related with this topic have been reported and the innovation is not new enough. This work can not be recommended for publication because of the following problems.
1. In the Introduction section, the selection reason of S as vacancies for improving the water oxidation activity of CdIn_2_S_4_ photoanode is not solidly introduced. Several typical works related to the S vacancies introduced photoanodes have been reported in recent years.
2. To introduce the S vacancies into photoanode, the CdIn_2_S_4_ was annealed with Ar/H_2_ flow at 500^o^C. The Ar/H_2_ possesses reducibility, will the photoanode be reduced? The XPS date about S and O should be provided.
3. The author claimed when the photoanode was annealed at 500^o^C, the In_2_O_3_ appeared, but the annealing flow is Ar/H_2_, why did oxides appear?
4. The dark photocurrent measured in 0.35 M Na_2_SO_3_ and 0.25 M Na_2_S mixed solution (pH =12.5) should be provided.
5. After the long-term J-t test, the authors should provide the XPS data to indicate its robust photoelectrochemical stability.
6. The instability of photoanode is due to the photocorrosion caused by holes collected in the photoanode surface, thus the long-term J-t test must be measured in Na_2_SO_4_.
7. The η_seperation_ and η_injection_ was measured in the electrolyte solution with and without Na_2_SO_3_ as a hole scavenger in the Figure 2a, why the J-V plots was measured in 0.35 M Na_2_SO_3_ and 0.25 M Na_2_S mixed solution (pH =12.5).

Reviewer #3 (Remarks to the Author):

Reviewer's remarks on the manuscript "Highly active deficient ternary sulfide photoanode for photoelectrochemical water splitting"

The manuscript reports on photoelectrocatalytic activity of spinel CdIn_2_S_4_ photoanodes with artificially introduced sulfur vacancies in wster splitting. The authors report unprecedently high light-to-current and light-to-hydrogen conversion efficiencies along with absolutely unexpected stability of such S-deficient material both in the splitting of pure water where typically strong photocorrosion of sulfides occur and in solutions containing sulfide ions that can eliminate the surface vacancies. The authors provided very deep experimental and calculational background explaining this unusual stability of the S-deficient CdIn_2_S_4_ photoanodes due to the presence of undercoordinated Cd and IN atoms (that is S vacancies).
The spinel CdIn_2_S_4_ is only occasionally studied as a photocatalyst and even rarely - as a photoelectrocatalyst and therefore the material as well as the approach for generation of the sulfur vacancies can be considered as novel. The authors then performed extensively deep characterization of the photoactive phase including probably all imaginable (among appropriate ones) physical and physio-chemical methods that can be applied to extract information on such materials, including EPR verification of vacancies and their direct observation through HAADF STEM. Also, the electrochemical properties of the photoanode-electrolyte system are studied deeply and comprehensively. The authors also addressed the issues of the kinetics and mechanism of the interfacial hole transfer on the surface of S-deficient CdIn_2_S_4_ and provided a deep insight into this process which undoubtedly contributes to our knowledge of the photoanodic processes on sulfide semiconductors and will inspire deeper studies of both this particular semiconductor and other possible ternary spinel compounds as well as future searches for the most favorable co-catalysts of water reduction and oxidation with such materials. The paper is logically built and written with acceptable language, the figures are excellent and rationally distributed between the main text and SI. No particular critical comments appeared during the analysis of the manuscript. Therefore, it can be recommended for the publication in the present form.

**Responses to the reviewer's comments**

Reviewer #1 (Remarks to the Author):

This manuscript investigates CdIn_2_S_4_, a ternary metal sulfide, for its application as photoanode and its photoelectrochemical performance impact of introducing sulfur vacancies. They conduct experimental characterization supported with theoretical calculations. A detailed study experimental study shows that ternary sulfide is a potential candidate as a photoanode for photoelectrochemical water splitting. The surface vacancies, due to missing S, results in improved photocurrent.
The paper has a good quality of research, with substantial experimental details. To conclude, the content of the paper is publishable with edits.

**Response:** Thank you for the constructive comments, which are very helpful for us to improve the quality of this article.
There are a few suggestions to improve the paper:
1. Theoretical investigation is missing basic information, like how the calculations were set up. Please consider including simulation details.

**Response:** Thanks for this good suggestion. The details for the parameters set in the DFT calculations have been supplemented in the revised manuscript. The first-principle calculations corrected by on-site Coulomb interaction were carried out with the Vienna ab initio simulation package (VASP). The interaction between ions and valence electrons was described using the projector augmented wave (PAW) potentials, and the exchange-correction function was treated using the generalized gradient approximation (GGA) in the Perdew-Burke-Ernzerhof for solid (PBEsol) form. Interactions between the valence electrons and the ion core are represented in 4d^10^5S^2^, 4d^10^5s^2^5p^1^, and 3s^2^3p^4^ orbitals for Cd, In and S, respectively. The wave functions were expanded in a plane wave basis with an energy cutoff of 500 eV, which was high enough to ensure that no Pulay stresses occur within the cell during geometry relaxation. The effective *U-J* values of 2.1 and 1.9 were employed to account for the strong on-site Coulomb interaction of Cd and In atoms, respectively. The Brillouin zone was sampled by a Γ-centered method, and a K-points resolved value of 0.02 was employed for all the geometries optimization, which was set to 0.01 for the density of states calculations. The Fermi level was slightly broadened using Fermi-Dirac smearing of 50 meV. For all the calculations, the convergence criteria for the electronic and ionic relaxation are 10^−5^ eV and 0.02 eV/Å, respectively. (Please see the revision in Page 28)

2. The way details of the experiment set up are described in the manuscript, they should include technical details regarding simulations.

**Response:** Thanks for this kind suggestion. Three theoretical parts are performed to illustrate the surface and electronic structures of CdIn_2_S_4_, that is, surface energy, the formation energy of sulfur vacancy, and surface OER mechanism. The surface energy calculations are performed to further verify the HAADF-STEM image, and more importantly, to reveal the surface atomic structure for the following sulfur vacancy study as well as surface OER mechanism. The details for simulations, especially to the model construction, have been supplemented as follows.

**Details for surface energy calculation**: Based on the BFDH theory and the experimental morphology of CdIn_2_S_4_ (Figure1E), we constructed the geometry of CdIn_2_S_4_, which consisted of {100}, {110}, and {$0\bar{1}1$} family of crystal planes. Given the effect of truncated atoms on the surface energy, (100), (110), and ($0\bar{1}1$) crystal planes with variable truncated atoms were taken into account. The symmetrical slab models with a vacuum thickness of 30 Å were adopted to simulate the geometries of possible exposed planes (Figure R1).

**Figure R1.** Slab models constructed for (100), (110), and ($0\bar{1}1$) crystal planes of CdIn_2_S_4_.

The surface energy was calculated according to the following equation:

$\gamma\text{=}\frac{1}{2A}(E_{\mathrm{s}\text{lab}}^{\mathrm{relax}}-\text{n}_{\text{Cd}}\mu_{\text{Cd}}\text{-n}_{\text{In}}\mu_{\text{In}}\text{-n}_{\text{S}}\mu_{\text{S}})$ (1)

Where *A* was the surface area,$E_{\mathrm{s}\text{lab}}^{\mathrm{relax}}$was the total energy of the relaxed slab, and *n*_Cd_, *n*_In_, and *n*_S_ were the numbers of Cd, In and S involved in the slab model, *μ*_Cd_*, μ*_In_*,* and *μ*_S_ were the atomic chemical potential of Cd, In and S atoms, respectively. The factor of 1/2 corresponded to two equivalent surfaces in the slab models. Detailed calculation processes were supplemented in supporting information.

The surface energy is highly relevant to the chemical potentials of *μ*_Cd_*, μ*_In_*,* and *μ*_S._ Therefore, we investigate the upper and lower boundary of *μ*_Cd_*, μ*_In_*,* and *μ*_S,_ as revealed in the supporting information. Given the excessive thioacetamide in the experimental condition which indicates the S rich in chemical potential, the surface energies are the average of the results calculated in condition C and D. As shown in Table S3, the exposed surface with In and S as truncated atoms is more stable in (0$\bar{1}$1), which were in good agreement with the HAADF-STEM image and provided the atomic structure of (0$\bar{1}$1) for simulating the surface OER process.

**Details for formation energy of sulfur vacancy calculation**: To illustrate the most feasible exposed plane for the surface sulfur vacancy formation, slabs models with vacuum thickness of 15 Å were constructed based on the above thermodynamically favorable surface structure, as shown in Figure R2. To minimize the effect of spurious electrostatic interactions in charged defect calculations due to the periodic cell approximation, a 2 × 2 periodic surfaces were employed to ensure the distance of the sulfur vacancies in adjacent cells over 10 Å. The bottom atoms were saturated with H and pseudo-potential 0.5H to remove the dangling bonds. The possibility of production of the surface sulfur vacancy in (100), (110), and ($0\bar{1}1$) crystal planes was evaluated by the formation energy which is estimated as,

 (2)

Where$E_{\mathrm{tot}}(X^{\mathrm{q}})$ and$E{}_{\mathrm{tot}}(\mathrm{bulk})$ were the total energies of the defect *X* with charge *q* and bulk, respectively. *n_i_* was the number of atoms being added to (*n*_i_ *>* 0) and/or removed from (*n*_i_ < 0) in the perfect-crystal supercell, and *μ*_i_ was the atomic chemical potential. *E*_VBM_ was the energy of the valence-band maximum (VBM), and *E*_F_ was the Fermi level measured from the VBM, varying in the range of the bandgap *E*_g_.

**Figure R2.** Slab models constructed for the surface S vacancy.

**Details for surface OER mechanism calculation**: The slab models of ($0\bar{1}1$) planes with In and S as truncated atoms were constructed to elucidate the potential-determining step in surface OER process by calculating the evolution of OH*, O*, and OOH* intermediates on CdIn_2_S_4_ and deficient CdIn_2_S_4_. As shown in Figure S3, the slab models consisted of eight atomic layers and a vacuum thickness of 15 Å. Similarly, the bottom atoms were saturated with H and pseudo-potential 0.5H to remove the dangling bonds. The calculation details for surface OER mechanisms were supplemented in supporting information.

**Figure R3.** The optimized geometries of OH*, O*, and OOH* intermediates absorbing on CdIn_2_S_4_-(0$\bar{1}$1) and *V*_s_-CIS-(0$\bar{1}$1).

3. From TDOS calculations of Fig. S21 it seems that S vacancies induce additional trap states in the bandgap. It might be useful to see references seeing such effect (either experimentally or theoretically). Also, explain how that leads to an improved photocurrent.

**Response:** Thanks for your good question. What your concern is correct. The trap states in the bandgap usually exhibit two kinds of effects: (1) the trap states extend the light-responsive region by accommodating the photons whose energies are lower than the bandgap, resulting in the improved photocurrent^1^; (2) the trap states act as a separation center for the photoinduced electron-hole pair and enhance the photocurrent^2^. Herein, we consider the trap states induced by S vacancies mainly act as separation centers. Firstly, light absorption edge (Fig. 3A) and the photon absorption rate (Fig. S15) of *V*_s_-CIS-500 do not reveal much enhancement relative to that of CdIn_2_S_4_, which illustrates that importing S vacancies does not improve much light-harvesting and *J*_abs_ is not the crucial factor for the boosted photocurrent density. Secondly, the increased surface photovoltage value of *V*_s_-CIS-500 directly evidence the enhancement of photoinduced charge separation. Thirdly, as shown in Figure S25, the shallow trap states mainly consist of S 2p orbitals. According to the DFT calculation, S vacancy with 2+ charge state has the lowest formation energy. Therefore, the surface S vacancy becomes positively charged $\mathrm{S}_{\mathrm{vac}}^{2+}$ and donates free carriers to the conduction band of CdIn_2_S_4_ as in other references^3,4^, resulting in the significant increase of the charge carrier density at the surface region, which is in good agreement with the Mott-Schottky results. Recent research proves that increasing the carrier density provides an effective method to shorten the depletion width and enhance the band bending for hole collection^5^. Herein, the conduction band edge in S-deficient CdIn_2_S_4_ photoanode is indeed negatively shifted relative to that of the CdIn_2_S_4_ photoanode, as shown in Figure 4c, which illustrates the sharper band bending in S-deficient CdIn_2_S_4_ photoanode and realizes an enhanced improvement in charge separation. Therefore, we consider the effect of surface S vacancy on the charge separation is responsible for the improved photocurrent. (Please see the revision in Page 16)

Reviewer #2 (Remarks to the Author):
In this manuscript, Wang et.al prepared and investigated the novel deficient ternary metal sulfide (CdIn_2_S_4_) as photoanodes for water oxidation. Through mild annealing treatment (Ar/H_2_, 500^o^C), the surface S vacancies were introduced to the CdIn_2_S_4_ photoanode. Higher photocurrent was observed on the photoanode relative to the pristine CdIn_2_S_4_ thin film. They attributed the enhanced activity of photoanode to the effect of surface S vacancies on the interfacial charge separation and transfer kinetics. They also demonstrated the restrained surface states distribution and the transformation of active sites after introducing surface S vacancies. However, many works related with this topic have been reported and the innovation is not new enough. This work can not be recommended for publication because of the following problems.

**Response:** Thanks for the reviewer's comments. The metal sulfides are have long been the topic of research, especially photocatalytic applications due to their suitable electronic bandgaps, exposed active sites, diverse and adjustable chemical structures. However, their applications, especially for the ternary metal sulfides CdIn_2_S_4_, in the PEC water splitting, are rarely reported. The CdIn_2_S_4_ employed as photoelectrocatalyst are rationally chosen according to the following reasons: (1) its n-type semiconductor characteristic as well as proper bandgap and band positions; (2) the soft 4d^10^ orbitals of In^3+^ are easily tuned, which facilitates the regulation of its intrinsic band structure^6^; (3) the Cd^2+^ and In^3+^ ions are separately located at the centers of tetrahedral and octahedral coordination geometry in spinel CdIn_2_S_4_, which ensures its structural stability. Based on the above analysis, we consider CdIn_2_S_4_ may be a good candidate for novel photoanode material. Indeed, there are a few excellent works related to the topic of the effect of S vacancies on the final photoelectrochemical properties of the metal sulfide photoanodes^7-10^, which also gives us many references and inspirations. However, employing ternary CdIn_2_S_4_ as photoelectrocatalyst has not been reported before and can be considered as novel. Moreover, in this manuscript, we have investigated the charge transfer kinetics and mechanism of the interfacial hole transfer on the surface of S-deficient CdIn_2_S_4_ in detail by experiments and theoretical simulations. Meanwhile, the S-deficient CdIn_2_S_4_ reveals comparable PEC performance to the other up-to-date promising photoanodes, such as BiVO_4_, Fe_2_O_3,_ and Ta_3_N_5_(TaON), which not only confirms the feasibility of employing CdIn_2_S_4_ as photoanode but also can inspire more excellent works based on CdIn_2_S_4_ and other sulfide-based photoanodes. We have made a significant revision based on the review's questions and suggestions. We will be grateful if the reviewer can consider this revised manuscript.

1. In the Introduction section, the selection reason of S as vacancies for improving the water oxidation activity of CdIn_2_S_4_ photoanode is not solidly introduced. Several typical works related to the S vacancies introduced photoanodes have been reported in recent years.

**Response:** Thanks for your good question. We have consulted the recent works related to the S vacancies and made a revision in the introduction part to illustrate the reason for introducing S vacancies to improve the water oxidation activity of the CdIn_2_S_4_ photoanode. These related works give us much inspiration in the materials synthesis, physicochemical characterizations, and mechanism discussions about S-deficient CdIn_2_S_4_. In this manuscript, the reasons for introducing S vacancies are three parts: (1) The trap states introduced by S vacancies maybe serve as separation centers for photoexcited electron-hole pairs^2^; (2) The intrinsic charge carrier density of the CdIn_2_S_4_ photoanode is low. As in the other reported metal sulfide semiconductors,^11-12^ S vacancies in CdIn_2_S_4_ could significantly increase the charge density. Recent research proves that increasing the carrier density provides an effective method to shorten the depletion width and enhance the band bending for hole collection^13^; (3) The charge may accumulate on the adjacent Cd and In atoms after introducing S vacancy as in other report^14^, which turns the Cd and In atoms into be active sites for chemisorption of the intermediates during the oxygen evolution reaction and facilitates the surface water oxidation kinetics. Based on the above reasons, we try to introduce S vacancy and investigate its effect on charge separation and transfer kinetics as well as the surface states distribution on the deficient CdIn_2_S_4_ photoanode. We have revised this part to illustrate the reasons for introducing S vacancies, and corresponding references have been cited. (Please see the revision in Page 4)

2. To introduce the S vacancies into photoanode, the CdIn_2_S_4_ was annealed with Ar/H_2_ flow at 500^o^C. The Ar/H_2_ possesses reducibility, will the photoanode be reduced? The XPS data about S and O should be provided.

**Response:** Thanks for your question. As shown in Figure R4a-b, no structural and morphological changes for the CdIn_2_S_4_ photoanode thermal treated at 500°C under H_2_/Ar mixed gas flux (1:10 vol/vol, 100 mL/min) atmosphere, which illustrates the photoanode is not reduced under H_2_/Ar except for introducing surface S vacancies as discussed in the manuscript. To further demonstrate the structural stability of the CdIn_2_S_4_ photoanode under the H_2_/Ar atmosphere, thermogravimetric (TG) analysis is performed under Ar/H_2_ atmosphere (1:10 vol/vol, 100 mL/min) with a heating rate of 5°/min. As shown in Figure R4c, only a small portion of weight loss for the CdIn_2_S_4_ photoanode appears even the temperature rises to 500°C (1.03% weight loss), which also illustrates the photoanode is not reduced at 500°C under Ar/H_2_ atmosphere during thermal treatment. The XPS spectra of S 2p orbital are supplemented in Figure R4d, which shifts to a higher binding energy position and indicates the formation of S vacancies in CdIn_2_S_4_ as in other articles. The XPS spectra of O 1s orbital are also supplemented, as shown in Figure R4e. The peak position of O 1s in *V*_s_-CIS-500 is nearly identical to that in pristine CdIn_2_S_4_, which illustrates the existence form of O should be surface hydroxyls. To further confirm this deduction, the Ar plasma treatment is employed to dispose of the surface of CdIn_2_S_4,_ and *V*_s_-CIS-500 samples, which reveals that the peak positions of O 1s disappear (Figure R4f). (Please see the revision in Page 5 and supporting information)

**Figure R4**. (a) XRD patterns and (b) SEM images of CdIn_2_S_4_ and *V*_s_-CIS-500. (c) TGA and DSC curves of CdIn_2_S_4_ under Ar/H_2_ atmosphere (1:10 vol/vol, 100 mL/min) with a heating rate of 5°/min. (d) Core-level XPS spectra of S 2p orbital. (e) XPS full spectrum for CdIn_2_S_4_ and *V*_s_-CIS-500, and (f) XPS spectra of O1s for CdIn_2_S_4_ and *V*_s_-CIS-500, and samples after Ar plasma treatment.
3. The author claimed when the photoanode was annealed at 500^o^C, the In_2_O_3_ appeared, but the annealing flow is Ar/H_2_, why did oxides appear?

**Response:** Thanks for your good question. After we carefully analyze the synthesis condition and experimental results, we conclude that the appearance of the In_2_O_3_ phase at 550° C may be caused by the air leakage in the tube furnace. We change another tube furnace and carefully check the airtightness, and reprepare the sample at 550° C. As shown in Figure R5a, except for CdIn_2_S_4_, the impurity phase of CdS appears due to the thermal decomposition of CdIn_2_S_4_. The ESR spectrum and the current density-voltage plot for *V*_s_-CIS-550 are also measured to examine the correlation between the intensities of the ESR signal and the photocurrent density. As shown in Figure R5b-c, the *V*_s_-CIS-550 photoanode reveals a relatively weaker intensity and inferior photocurrent, which illustrates that the enhanced photocurrent is indeed caused by the surface S vacancies and *V*_s_-CIS-500 possesses the optimal PEC performance. (Please see the revision in Page 7 and supporting information)

**Figure R5.** (a) XRD patterns of *V*_s_-CIS-500 and *V*_s_-CIS-550. (b) ESR spectra. (c) Current density-voltage plots for as-prepared products measured in 0.35 M Na_2_SO_3_ and 0.25 M Na_2_S mixed solution (pH = 12.5) under AM 1.5G (100 mW cm^-2^) illumination with a scan rate of 10 mV s^-1^.

4. The dark photocurrent measured in 0.35 M Na_2_SO_3_ and 0.25 M Na_2_S mixed solution (pH =12.5) should be provided.

**Response:** Thanks for your question. The dark photocurrents of photoanodes have been supplemented as revealed in Figure R6a, and the ABPE values (Figure R6b) derived from the current-voltage plots are also revised. (Please see the revision in Page 11)

**Figure R6**. (a) Current-voltage *(J-V*) plots measured in 0.35 M Na_2_SO_3_ and 0.25 M Na_2_S mixed solution (pH =12.5) under dark and AM 1.5G, 100 mW cm^-2^ illumination with a scan rate of 10 mV s^-1^. (b) ABPE measured with a two-electrode cell for water splitting in 0.35 M Na_2_SO_3_ and 0.25 M Na_2_S mixed solution (pH = 12.5) under dark and AM 1.5G, 100 mW cm^-2^ illumination.
5. After the long-term J-t test, the authors should provide the XPS data to indicate its robust photoelectrochemical stability.

**Response:** Thanks for your good question. The XPS spectra of *V*_s_-CIS-500 photoanode post-PEC test for 15 h in 0.35 M Na_2_SO_3_ and 0.25 M Na_2_S mixed solution are supplemented to illustrate the PEC stability further. The peak positions for Cd and In atoms in *V*_s_-CIS-500 photoanode post-PEC test for 15 h are nearly identical to those in pristine *V*_s_-CIS-500 photoanode (Figure R7a-b), while the S 2p orbital shifts to higher binding energy for ca. 0.08 eV (Figure R7c) and peaks that assigned to elemental sulfur appear (Figure R7d), which illustrates that partial surface S in *V*_s_-CIS-500 photoanode is oxidated, and suggests that the surface of *V*_s_-CIS-500 require OECs (oxygen evolution catalysts) to form a stable *V*_s_-CIS-500/OEC interface to achieve long-term stability for photo-oxidation of water. (Please see the revision in Page 11 and supporting information)

**Figure R7**. Core-level XPS spectra comparison of pristine *V*_s_-CIS-500 photoanode and the photoanode post-PEC test for 15 h in 0.35 M Na_2_SO_3_ and 0.25 M Na_2_S mixed solution. (a) Cd 3d, (b) In 3d, and (c-d) S 2p.

6. The instability of photoanode is due to the photocorrosion caused by holes collected in the photoanode surface, thus the long-term J-t test must be measured in Na_2_SO_4_.

**Response:** Thanks for your good question. What your concern is correct. The instability of the photoanodes is mainly caused by the surface oxidation of photoinduced holes during the PEC process. Given that the surface oxidation kinetics of sulfite is much faster than water, we employed 0.35 M Na_2_SO_3_ and 0.25 M Na_2_S mixed solution to evaluate the long-term stability of the *V*_s_-CIS-500 photoanode, which is also the general measurement condition as in other metal-sulfide based photoanodes.^15-17^ Besides, we have supplemented the long-term *J-t* test of the *V*_s_-CIS-500 photoanode in the 0.5 M Na_2_SO_4_ solution. The *V*_s_-CIS-500 photoanode reveals an inferior current-voltage response in the 0.5 M Na_2_SO_4_ solution (Figure R8a), and the photocurrent density quickly decreases in 1 h continued operation (Figure R8b), which also illustrates that further surface modification of the *V*_s_-CIS-500 photoanode is necessary to stabilize the *V*_s_-CIS-500/electrolyte interface to achieve long-term stability. (Please see the revision in Page 11 and supporting information)

**Figure R8**. (a) photocurrent-potential curves of the *V*_s_-CIS-500 photoanode under chopped light illumination in 0.5 M Na_2_SO_4_ solution and 0.35 M Na_2_SO_3_ and 0.25 M Na_2_S mixed solution. (b) Long-term stability tests of the *V*_s_-CIS-500 photoanode in 0.5 M Na_2_SO_4_ solution under AM 1.5G, 100 mW cm^-2^ illumination.

7. The η_seperation_ and η_injection_ were measured in the electrolyte solution with and without Na_2_SO_3_ as a hole scavenger in the Figure 2a, why the J-V plots were measured in 0.35 M Na_2_SO_3_ and 0.25 M Na_2_S mixed solution (pH =12.5).

**Response:** Thanks for your good question. We have made a mistake in labeling the subscript of the equations for calculating the values of *η*_seperation_ and *η*_injection_ (eq 6-7). These values were also measured in 0.35 M Na_2_SO_3_ and 0.25 M Na_2_S mixed solution (pH =12.5), which serves as hole scavengers and ensures the hole injection efficiency approaching 100% due to the rapid hole consumption rate. The measurement condition of employing 0.35 M Na_2_SO_3_ and 0.25 M Na_2_S mixed solution as the electrolyte in the J-V plots is an optimal result ^18^, as referred to in the other article. Therefore, we adopt this mixed electrolyte and obtain the photocurrent at 1.23 V vs. RHE to compare with the other up-to-date metal sulfide-based photoanodes, as revealed in Table S1. Besides, we also have measured the J-V plots in the Na_2_SO_3_ electrolyte, as shown in Figure R9, which indeed reveals inferior PEC performance. We have revised the equations in this manuscript. (Please see the revision in Page 25-26)

**Figure R9.** Current-voltage *(J-V*) plots measured in 0.35 M Na_2_SO_3_ and 0.25 M Na_2_S mixed solution, and 0.6 M Na_2_SO_3_ under AM 1.5G, 100 mW cm^-2^ illumination with a scan rate of 10 mV s^-1^.

Reviewer #3 (Remarks to the Author):

Reviewer's remarks on the manuscript "Highly active deficient ternary sulfide photoanode for photoelectrochemical water splitting"

The manuscript reports on photoelectrocatalytic activity of spinel CdIn_2_S_4_ photoanodes with artificially introduced sulfur vacancies in water splitting. The authors report unprecedently high light-to-current and light-to-hydrogen conversion efficiencies along with absolutely unexpected stability of such S-deficient material both in the splitting of pure water where typically strong photocorrosion of sulfides occur and in solutions containing sulfide ions that can eliminate the surface vacancies. The authors provided very deep experimental and calculational background explaining this unusual stability of the S-deficient CdIn_2_S_4_ photoanodes due to the presence of undercoordinated Cd and In atoms (that is S vacancies).
The spinel CdIn_2_S_4_ is only occasionally studied as a photocatalyst and even rarely - as a photoelectrocatalyst and therefore the material as well as the approach for generation of the sulfur vacancies can be considered as novel. The authors then performed extensively deep characterization of the photoactive phase including probably all imaginable (among appropriate ones) physical and physio-chemical methods that can be applied to extract information on such materials, including EPR verification of vacancies and their direct observation through HAADF STEM. Also, the electrochemical properties of the photoanode-electrolyte system are studied deeply and comprehensively. The authors also addressed the issues of the kinetics and mechanism of the interfacial hole transfer on the surface of S-deficient CdIn_2_S_4_ and provided a deep insight into this process which undoubtedly contributes to our knowledge of the photoanodic processes on sulfide semiconductors and will inspire deeper studies of both this particular semiconductor and other possible ternary spinel compounds as well as future searches for the most favorable co-catalysts of water reduction and oxidation with such materials. The paper is logically built and written with acceptable language, the figures are excellent and rationally distributed between the main text and SI. No particular critical comments appeared during the analysis of the manuscript. Therefore, it can be recommended for the publication in the present form.

**Response:** Thanks for such positive comments.

**References**

1.	Ran J, Ma TY, Gao G, Du X-W, Qiao SZ. Porous P-doped graphitic carbon nitride nanosheets for synergistically enhanced visible-light photocatalytic H_2_ production. *Energy Environ. Sci.* **8**, 3708-3717 (2015).

2.	Jiao X*, et al.* Defect-mediated electron-hole separation in one-unit-cell ZnIn_2_S_4_ layers for boosted solar-driven CO_2_ reduction. *J. Am. Chem. Soc.* **139**, 7586-7594 (2017).

3.	Wang S, Chen P, Bai Y, Yun J-H, Liu G, Wang L. New BiVO_4_ dual photoanodes with enriched oxygen vacancies for efficient solar-driven water splitting. *Adv. Mater.* **30**, 1800486 (2018).

4.	Feng S*, et al.* Enriched surface oxygen vacancies of photoanodes by photoetching with enhanced charge separation. *Angew. Chem. Inter. Ed.* **59**, 2044-2048 (2020).

5.	Tian Z*, et al.* Novel Black BiVO_4_/TiO_2−x_ photoanode with enhanced photon absorption and charge separation for efficient and stable solar water splitting. *Adv. Energy Mater.* **9**, 1901287 (2019).

6.	Walter MG*, et al.* Solar Water Splitting Cells. *Chemical Reviews* **110**, 6446-6473 (2010).

7.	Meng L, Wang S, Cao F, Tian W, Long R, Li L. Doping-induced amorphization, vacancy, and gradient energy band in SnS_2_ nanosheet arrays for improved photoelectrochemical water splitting. *Angew. Chem. Inter. Ed.* **58**, 6761-6765 (2019).

8.	Giri B*, et al.* Balancing light absorption and charge transport in vertical SnS_2_ nanoflake photoanodes with stepped layers and large intrinsic mobility. *Advanced Energy Materials* **9**, 1901236 (2019).

9.	Fu Y*, et al.* Phase-modulated band alignment in CdS nanorod/SnS_x_ nanosheet hierarchical heterojunctions toward efficient water splitting. *Adv. Funct. Mater.* **28**, 1706785 (2018).

10.	Tian Z*, et al.* Enhanced charge carrier lifetime of TiS_3_ photoanode by introduction of S_2_^2−^ vacancies for efficient photoelectrochemical hydrogen evolution. *Adv. Funct. Mater.*, 2001286 (2020).

11.	Sun X*, et al.* Enhanced superoxide generation on defective surfaces for selective photo-oxidation. *J. Am. Chem. Soc.* **141**, 3797-3801 (2019).

12.	Zhang S*, et al.* MoS_2_ quantum dot growth induced by S vacancies in a ZnIn_2_S_4_ monolayer: Atomic-level heterostructure for photocatalytic hydrogen production. *ACS Nano* **12**, 751-758 (2018).

13.	Li C*, et al.* Surviving high-temperature calcination: ZrO_2_-induced hematite nanotubes for photoelectrochemical water oxidation. *Angew. Chem. Inter. Ed.* **56**, 4150-4155 (2017).

14.	Li X*, et al.* Selective visible-light-driven photocatalytic CO_2_ reduction to CH_4_ mediated by atomically thin CuIn_5_S_8_ layers. *Nat. Energy* **4**, 690-699 (2019).

15.	Wang R, Wang L, Zhou Y, Zou Z. Al-ZnO/CdS Photoanode modified with a triple functions conformal TiO_2_ film for enhanced photoelectrochemical efficiency and stability. *Appl. Catal. B-Environ.* **255**, 117738 (2019).

16.	Simon T*, et al.* Redox shuttle mechanism enhances photocatalytic H2 generation on Ni-decorated CdS nanorods. *Nat. Mater.* **13**, 1013-1018 (2014).

17.	Zheng X-L*, et al.* Strongly coupled nafion molecules and ordered porous CdS networks for enhanced visible-light photoelectrochemical hydrogen evolution. *Adv. Mater.* **28**, 4935-4942 (2016).

18.	Bao N, Shen L, Takata T, Domen K. Self-templated synthesis of nanoporous CdS nanostructures for highly efficient photocatalytic hydrogen production under visible light. *Chem. Mater.* **20**, 110-117 (2008).

**Reviewer comments, second version:**

Review #1

This is a revised submission of the manuscript NCOMMS-20-00744. The manuscript investigates CdInS4, a ternary metal sulfide, for its application as photoanode and its photoelectrochemical performance impact of introducing sulfur vacancies. They conduct experimental characterization supported with theoretical calculations.

A detailed study experimental study shows that ternary sulfide is a potential candidate as a photoanode for photoelectrochemical water splitting. The surface vacancies, due to missing S, results in improved photocurrent. The paper has a good quality of research, with substantial investigations and details of the approaches.

The revision addresses all the previous concerns satisfactorily. To conclude, the content of the paper is publishable without any further edits.

Reviewer #2 (Remarks to the Author):

This revised version has addressed the comments well, and can be accepted

**Author rebuttal, second version:**

**Reviewer comments, third version:**

**Author rebuttal, third version:**
